# The Current State of Molecular Testing in the BRAF-Mutated Melanoma Landscape

**DOI:** 10.3389/fmolb.2020.00113

**Published:** 2020-06-30

**Authors:** Irene Vanni, Enrica Teresa Tanda, Francesco Spagnolo, Virginia Andreotti, William Bruno, Paola Ghiorzo

**Affiliations:** ^1^Genetics of Rare Cancers, IRCCS Ospedale Policlinico San Martino, Genoa, Italy; ^2^Genetics of Rare Cancers, Department of Internal Medicine and Medical Specialties, University of Genoa, Genoa, Italy; ^3^Medical Oncology IRCCS Ospedale Policlinico San Martino, Genoa, Italy

**Keywords:** melanoma, *BRAF* mutation, targeted therapy, liquid biopsy, molecular techniques, NGS

## Abstract

The incidence of melanoma, among the most lethal cancers, is widespread and increasing. Metastatic melanoma has a poor prognosis, representing about 90% of skin cancer mortality. The increased knowledge of tumor biology and the greater understanding of the immune system role in the anti-tumor response has allowed us to develop a more rational approach to systemic therapies. The discovery of activating *BRAF* mutations in half of all melanomas has led to the development of molecularly targeted therapy with BRAF and MEK inhibitors, which dramatically improved outcomes of patients with stage IV BRAF-mutant melanoma. More recently, the results of clinical phase III studies conducted in the adjuvant setting led to the combined administration of BRAF and MEK inhibitors also in patients with resected high-risk melanoma (stage III). Therefore, *BRAF* mutation testing has become a priority to determine the oncologist's choice and course of therapy. In this review, we will report the molecular biology-based strategies used for *BRAF* mutation detection with the main advantages and disadvantages of the most commonly used diagnostic strategies. The timing of such molecular assessment in patients with cutaneous melanoma will be discussed, and we will also examine considerations and approaches for accurate and effective *BRAF* testing.

## Introduction

The incidence of malignant melanoma has risen steadily during the last few decades, particularly in the Caucasian population (Siegel et al., 2017). According to GLOBOCAN (Global Cancer Observatory^1^), more than 287,723 new cases of melanoma of the skin (1.6% of all cancers) occurred worldwide in 2018, and ~60,712 deaths were reported. In Italy, a total of 13,000 cases were diagnosed in 2018, and a similar number was estimated for 2019 (NUMERI DEL CANCRO AIOM AIRTUM, January 2020^2^).

The etiology of melanoma is mostly related to mutagenic damages caused by the UVs with the involvement of many tumor suppressor genes and/or oncogenes (Hodis et al., 2012; Krauthammer et al., 2012; Akbani et al., 2015; Hayward et al., 2017). Indeed, cutaneous melanoma is characterized by a high prevalence of somatic mutations, both in the primary tumor and in the metastatic lesions, with a mean mutational burden over 20 mutations per megabase (Alexandrov et al., 2013; Akbani et al., 2015), one of the highest Tumor Mutation Burden (TMB) measurements among all solid tumors (Alexandrov et al., 2013; Zhang et al., 2016c). TMB is correlated with clinical response to cytotoxic T lymphocyte–associated antigen−4 blockade in advanced melanoma and with anti–programmed cell death protein−1 (PD-1) and/or PD-L1 blockade in melanoma (Snyder et al., 2014; Van Allen et al., 2015; Hugo et al., 2016; Johnson et al., 2016; Goodman et al., 2017; Cristescu et al., 2018). BRAF is a key element of the MAPK pathway, and it is physiologically activated by the binding between extracellular signals and their membrane receptor, typically a Tyrosine Kinases Receptor (TKR) (Lito et al., 2013). Activated BRAF kinase activates MEK, which, in turn, activates ERK, regulating cell proliferation and survival. An activating mutation in *BRAF*, with a constitutive activation of the kinase, is found in about 50% of cutaneous melanomas, mostly the *BRAF* V600E (Tate et al., 2019). On this basis, targeted therapies for the treatment of *BRAF* mutated, advanced, or metastatic melanoma were introduced in treatment protocols. First, BRAF inhibitors were approved as single agents (Queirolo and Spagnolo, 2017). Subsequently, the combination of BRAF inhibitors plus MEK inhibitors seemed to further improve the outcome in terms of Overall Survival (OS) among treated patients and combination therapy became the standard of treatment for BRAF mutant melanoma. Targeted therapies radically improved efficacy and survival outcomes among these patients, with a median OS of 25.9 months (Robert et al., 2019), whereas, historically, median survival with chemotherapy and bio-chemotherapy was only about 6 months (Korn et al., 2008).

More recently, between 2018 and December 2019, FDA and EMA released the approval for prescribing BRAF and MEK inhibitors in high-risk resected (stage III) melanoma patients (Spagnolo et al., 2019). Adjuvant treatment with combined BRAF and MEK inhibitors achieved a 53% decrease in the risk of relapse compared with placebo (Long et al., 2017).

The dramatic improvements in the outcomes in *BRAF*-mutated melanoma patients receiving BRAF and MEK inhibitors highlights the need for molecular assessment, which has become a necessary step to identify patients who are best suitable for targeted therapies or immunotherapies. However, despite several trials and studies having proven the efficacy of immuno checkpoint inhibitors (Moser et al., 2019; Pavlick et al., 2019) and targeted therapies in OS, no direct comparison has been performed so far, and the choice between the two therapeutic approaches in BRAF mutated melanoma is still debated. In this review, we will report the most commonly used *BRAF* diagnostic strategies for melanoma and the emerging techniques in the liquid biopsy field.

## Molecular Background

Over the past few decades, growing efforts have shown that tumors often show recurrent oncogenic mutations, amplifications, and rearrangements in the genes that drive cell to proliferation and survival. These alterations can occur in different genes considered “drivers” and can be mutually exclusive within the same tumor, such as *BRAF* and *NRAS* gene mutations. However, some studies showed *BRAF* and *NRAS* mutations in the same tumor samples, suggesting that these mutations are not mutually exclusive in melanoma but exhibit intra-tumoral heterogeneity (Sensi et al., 2006; Jovanovic et al., 2010).

**Figure 1:**
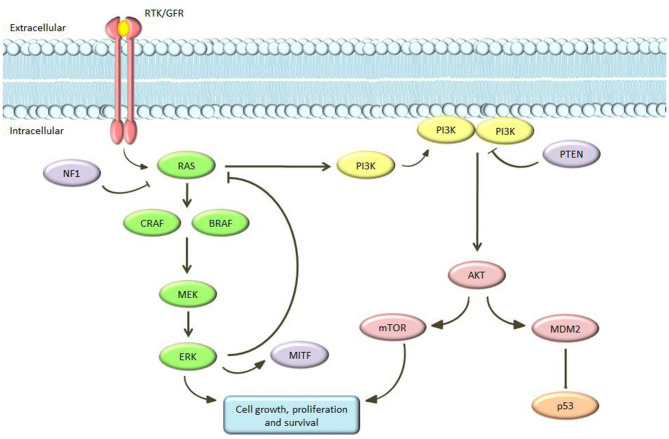
Schematic view of the MAPK and PI3K pathways.

The Cancer Genome Atlas (TCGA) database and other key sequencing studies has provided a comprehensive survey of the genetic landscape of cutaneous melanomas. Data published with TCGA Network, after the whole exome sequence analysis of 333 primary and/or metastatic melanoma patients, found that cutaneous melanomas could be classified into four genomic subgroups: mutant *BRAF*, mutant *NRAS*, mutant *NF1*, and triple-wild type (Berger et al., 2012; Furney et al., 2014; Akbani et al., 2015; Johansson et al., 2015; Hayward et al., 2017; Hintzsche et al., 2017; Lyu et al., 2017; Palmieri et al., 2018; Wilmott et al., 2019; Zhou et al., 2019). Critical signaling pathways in cutaneous melanoma are the RAS-RAF-MEK-ERK, PI3K/PTEN, and c-Kit pathways. The MAPK pathway physiologically plays a key role in the control of cell proliferation and cell differentiation (Dhillon et al., 2007). This pathway consists of several molecules that interact to each other in an on/off mechanism mediated by phosphorylation and dephosphorylation cycles. This signaling cascade induces the production of intracellular signals aimed to promote cellular growth, differentiation, and division (Figure 1). The transmembrane TKR/growth factor receptors (GFR) is the first molecule involved. The binding of this receptor to a growth factor results in the activation of RAS. RAS belongs to the GTPase family and acts as an on/off switch by activating or deactivating the entire downstream path (Gaestel, 2006). It activates the subsequent element in the cascade: RAF (a serine/threonine kinase with three isoforms—ARAF, BRAF, and CRAF—each of them with distinct characteristics in tissue distribution, kinase activity, and regulation) (Kwong and Chin, 2010; Matallanas et al., 2011). In turn, RAF phosphorylates and activates MEK (MEK1/2) and this process is repeated in successive phosphorylation steps ending with ERK activation. ERK ultimately acts by regulating the expression of a series of genes involved in proliferation, differentiation and survival. At the same time, activated ERK inhibits the MAPK pathway, acting as a negative feedback. Mutations of RAS, RAF, and MEK were described in a large number of tumors: for example, RAS mutations have been found in about 1/3 of all human tumors and in 15–20% of melanomas, and *BRAF* is mutated in 8% of human tumors and in about 40–50% of melanomas. Moreover, a 15% of melanomas shows mutations of *NF1* with loss of function. All of these genetic changes result in alterations of MAPK signaling functioning with uncontrolled cell proliferation (Wan et al., 2004).

The identification of *BRAF* mutations is crucial for personalized treatment of melanoma, being an important tool for diagnosis, treatment, predictor of patient outcomes, and may have an impact on prognosis (Barbour et al., 2014). Patients harboring activating mutations in *BRAF, NRAS*, and *KIT* genes could benefit of target treatment options. Despite this good purpose, to date, only *BRAF* mutations have FDA approved therapies in advanced cutaneous melanoma, whereas *KIT* mutations have the tyrosine kinase inhibitors Imatinib as off-label prescription. Based on recent ESMO Clinical Practice Guidelines, *BRAF* mutation testing is mandatory in patients with resectable or unresectable stage III or stage IV melanoma and is highly recommended in high-risk resected disease stage IIC patients (Michielin et al., 2019). In the metastatic setting, it is recommended to perform molecular analyses in metastatic sample, if available, because it represents the most recent lesion and it is often composed by a large majority of neoplastic cells. When a metastatic tissue sample is not available, the analyses may be performed on samples obtained from lymph node metastases or primary tumor since a high concordance of the *BRAF* status between primary melanomas and their metastatic lesions has been demonstrated (Colombino et al., 2012; Casula et al., 2016; Valachis and Ullenhag, 2017; Cormican et al., 2019; Pellegrini et al., 2020). Additionally, the NCCN clinical practice guidelines recommend *BRAF* mutation testing in patients with resectable and unresectable or metastatic melanoma to guide treatment decisions (NCCN Clinical Practice Guidelines in Oncology^3^)?

Mutational analysis is normally performed on Formalin-Fixed Paraffin-Embedded (FFPE) tissues, after removal of paraffin and DNA extraction with standardized protocols. Prior to molecular analysis, the sample must undergo enrichment, as the proportion of cancer cells should not be lower than 50% of the total. In case of melanomas arising on pre-existing nevi, particular care should be taken during the enrichment process to make sure that only melanoma cells are isolated from the tissue sample, as also melanocytic nevi can carry *BRAF* mutations. *BRAF* mutations occur quite often in melanomas, conferring to this kinase the ability to independently activate MEK, inducing its constitutive activation (Johnson and Sosman, 2015). However, mutations in *BRAF* are early and not sufficient to induce melanoma: there is a high frequency of these mutations in benign nevi (including congenital, intra-dermal, and dysplastic) (Pollock et al., 2003; Tschandl et al., 2013), and it seems that additional genetic events are necessary.

The discovery of *BRAF* mutation and its meaning occurred in 2002 and to date, about 300 *BRAF* mutations have been characterized, most in codon 600: this discovery paved the way for the development of new molecular-target drugs (Flaherty et al., 2012). Indeed, 37 to 60% of melanomas, typically those related to intermittent sun exposure damage, show a somatic mutation in *BRAF* (Curtin et al., 2005). Most *BRAF* mutations are missense variations that determine aminoacid substitution at valine 600. From 70, up to 88% of *BRAF* mutations are represented by V600E (valine to glutamic acid), but V600K (valine to lysine substitution) or V600D (valine to aspartic acid) and V600R (valine to arginine) are found in 5–12% and ≤ 5% of melanomas, respectively. (Rubinstein et al., 2010; Long et al., 2011; Lovly et al., 2012; Menzies et al., 2012). Recently, Yao et al. has further categorized *BRAF* mutations based on their MAPK pathway activation mechanisms into class-1 (high kinase activity involving codon 600), class-2 (high or intermediate kinase activity involving codons outside 600), and class-3 (impaired BRAF kinase activity) (Yao et al., 2017). Melanomas with *BRAF* class-1 (V600 mutations) respond well to current FDA-approved BRAF inhibitors (vemurafenib, dabrafenib, and encorafenib), as well as combined BRAF/MEK inhibitor therapy. On the contrary, melanomas with *BRAF* class-2 mutations (non-V600 mutations) do not respond to first-generation BRAF inhibitors, which are monomer selective, but they could be of benefit to MEK/ERK inhibitors as well as the BRAF inhibitor PLX8394 (Dahlman et al., 2012; Bowyer et al., 2014; Marconcini et al., 2017; Janku et al., 2018). These class II mutations can be further subdivided into class IIa within the activation segment (L597 and K601) and IIb within the glycine-rich region (G466 and G469) (Dankner et al., 2018). Finally, class III mutations (N581 and D594) have no kinase activity but facilitate RAS binding and CRAF activation (Yao et al., 2017).

To date, FDA approved only BRAF/MEK inhibitors alone or in combination in presence of *BRAF* V600 mutations. Three combinations of BRAF/MEK inhibitors (vemurafenib plus cobimetinib, dabrafenib plus trametinib, and encorafenib plus binimetinib) are currently available for *BRAF* V600E/K metastatic melanomas, and one combination (dabrafenib plus trametinib) has been recently approved in the adjuvant, Stage III setting (Long et al., 2017; Schvartsman et al., 2019).

## Assessment of *BRAF* Mutations

Several methods have been developed and are currently used for the detection of *BRAF* mutations, including Sanger sequencing, immunohistochemistry (IHC), pyrosequencing, mutation-specific Polymerase Chain Reaction (PCR) and mutation-specific real-time PCR, digital PCR (dPCR), High-Resolution Melting curve analysis (HRM), Matrix Assisted Laser Desorption Ionization-Time Of Flight Mass Spectrometry (MALDI-TOF MS; Sequenom), and Next-Generation Sequencing (NGS). The sensitivity and specificity of the most commonly used diagnostic methods are reported in Table 1.

**Table 1:** Sensitivity and specificity of the most commonly *BRAF* diagnostic techniques.

**Diagnostic technique**	**Sensitivity%**	**Specificity%**	**LOD%**
IHC	93–97	92–98	-
Sanger sequencing	80–93	100	20–25
Pyrosequencing	95–100	90–100	5–10
Real-time PCR based techniques	93–99.5	98–100	0.5–5
dPCR	100	95	0.001
HRM	87–99	96–99	5.0
MALDI-TOF MS	97.6	100	1–5
NGS	98	100	5

### Sanger Sequencing

Sanger sequencing has long been considered the reference method for the identification of acquired mutations in tumors. It requires a high percentage of tumor cells within the samples, which is not always possible in routine diagnostic testing. In this context, pathologists play a critical role in the triage of melanoma samples for molecular tests since they must estimate the cellularity of the tumor within the designated region to obtain sufficient material to ensure the correct analytical sensitivity of the test required. For this reason, it may be advisable perform macro-dissection by pathologists before mutation testing when tumor cell percentage is < 50%. Moreover, the sensitivity of Sanger sequencing for V600E detection is 92.5% (Anderson et al., 2012), meaning that, using this technique alone, 7.5% of patients potentially eligible for treatment with BRAF and MEK inhibitors would be missed. In this respect, three papers compared Sanger sequencing with the Cobas 4800 BRAF V600 Mutation Test (Roche Molecular Systems, Inc.), an FDA-approved real-time PCR assay to select patients with metastatic melanoma for treatment with the selective BRAF inhibitor vemurafenib or with cobimetinib plus vemurafenib combination (Lopez-Rios et al., 2013; Qu et al., 2013; Jurkowska et al., 2015). Qu et al. analyzed V600 mutations in 275 melanoma FFPE samples and displayed a higher sensitivity for Sanger sequencing than for Cobas 4800 test. The positive percent agreement and negative percent agreement for Sanger sequencing was 97.7 and 95.3%, respectively (Qu et al., 2013). Lopez-Rios et al. compared the Cobas BRAF Mutation Test with Sanger sequencing observing invalid results (DNA not amplified, difficult sequence interpretation, insufficient tumor content or DNA) in 8/116 specimens with Sanger vs. 0/232 with the Cobas BRAF test. The positive percent agreement and negative percent agreement was 97.7% and 95.3% for Sanger sequencing (Lopez-Rios et al., 2013). Finally, Jurkowska et al. examined *BRAF* mutations in 236 FFPE cutaneous melanoma lymph node metastases by Sanger sequencing tests and the Cobas® 4800 BRAF V600 Mutation Test. The study reported a very similar mutation detection rate between the two methods (60.9% for Sanger sequencing vs. 61.0% for Cobas 4800). Moreover, the sequencing demonstrated a superiority in the detection of mutations other than V600E but a higher susceptibility to DNA quality compared to Cobas 4800 test (Jurkowska et al., 2015). In general, the Limit Of Detection (LOD) of clinical assays by Sanger sequencing is ~20% to 25% mutant alleles. However, a lower percent mutant allele may be rarely detected, depending on the context of the targeted sequences (Pichler et al., 2009; Tsiatis et al., 2010). For these reasons, Sanger sequencing cannot be considered as a reference test but only as a screening or confirmation test (Spagnolo et al., 2015).

### Immunohistochemistry

To date, IHC with VE1 monoclonal antibody is the only available antibody-based test (Capper et al., 2012). This test was found to effectively detect V600E mutations (Colomba et al., 2013; Long et al., 2013). Recently, Vallée A et al. analyzed *BRAF* mutation in 60 metastatic melanoma tissues with BRAF IHC and Idylla^TM^ BRAF Mutation Assay, a real-time PCR assay. In this study, IHC yielded a final Predictive Positive Value (PPV) of 100% and a Negative Predictive Value (NPV) of 93% calculated with V600E mutated samples only (Vallée et al., 2019). In another study, the researchers reported a high concordance between allele-specific TaqMan assay and IHC in *BRAF* V600E detection analyzing 97 melanomas of 44 multiple primary melanoma patients. In particular, they reported a concordance of 88.7% (86/97) among the two techniques (Pellegrini et al., 2018). Discordant results were founded in 8/97 (8.2%) samples that showed weak or moderately positive VE1 immunostaining (Pellegrini et al., 2018). Several works suggest that we be careful in the case of presence of unclear weak staining with VE1 antibody (Busam et al., 2013; Ihle et al., 2014; Uguen et al., 2015). Moreover, Ihle et al. analyzed *BRAF* mutational status in 63 melanoma patients by HRM, pyrosequencing, allele specific PCR, NGS, and IHC reporting a cross-reactivity with no-V600E mutations (Ihle et al., 2014).

To perform this highly sensitive and specific test, only two tissue slides are required, and there is no need of specialized equipment. Other advantages of this technique are low costs and time, as results are obtained within 48 h. However, IHC has limitations derived from pre-analytical factors such as heterogeneously stained tumors, low tumor purity, necrotic tumor areas, and a suboptimal fixation condition (Capper et al., 2012; Dvorak et al., 2014; Fisher et al., 2014; Rapisuwon et al., 2016). In addition, staining interpretation is not always easy being subject to interpretation by the pathologist (Fisher et al., 2014). Moreover, the VE1 antibody is highly specific only for *BRAF* V600E, then IHC may be used as a cost-effective first-line method for *BRAF* V600E as part of a routine combination of methods (Colomba et al., 2013; Pearlstein et al., 2014; Spagnolo et al., 2015; Tetzlaff et al., 2015).

### Pyrosequencing

Pyrosequencing differs from Sanger methods for the detection of pyrophosphate release and the generation of light on nucleotide incorporation and not of chain termination with dideoxynucleotides. Pyrosequencing is commonly used to detect *BRAF* mutations and commercial kits are actually available for *BRAF* analysis on this platform, such as therascreen BRAF Pyro kit (Qiagen, Hilden, Germany) (Tan et al., 2008). In general, pyrosequencing is unable to accurately detect variants within >5 or 6 bp homopolymer and can be generate confusing patterns difficult to resolve without further investigation in the case of complex mutations. Moreover, pyrosequencing allows the analysis of single nucleotide polymorphisms or hotspot mutations in a single run since the length of sequence is usually <200 bp. However, compared with Sanger sequencing, pyrosequencing is a quantitative method, has a superior LOD (5% minor allele frequency), and is faster (Spittle et al., 2007). Compared to direct sequencing, pyrosequencing is a rapid and more sensitive method for quantifying the *BRAF* V600 mutations. Across different studies, pyrosequencing sensitivity and specificity for the detection of *BRAF* V600 mutations range from 90 to 100% and from 95 to 100%, respectively (Colomba et al., 2013; Ihle et al., 2014). Therefore, this technique may be used in those cases in which results provided by IHC and Sanger sequencing are uncertain (Spagnolo et al., 2015).

### Real-Time PCR-Based Techniques

Real-time PCR-based tests showed a higher sensitivity than Sanger sequencing, with specificity ranging from 88 to 100% depending on whether their design is specific for the V600 mutations. The FDA/CE-IVD-approved tests for *BRAF* mutations (Cobas® 4800 BRAF p.V600 mutation test and THxID®-BRAF) are both real-time PCR-based assays (http://www.fda.gov/companiondiagnostics). The main limitation of these approaches is that they are optimized for the most common *BRAF* mutations. In particular, THxID-BRAF kit was approved for the detection of V600E and K mutations and is validated for DNA input ranging from 10 to 350 ng/μl (bioMérieux Corporate Website^4^). On the contrary, the Cobas 4800 test is FDA approved only for V600E and has an analytical sensitivity of 95% for detecting the V600E mutation with a recommended DNA input of 125 ng total (125 ng/25 μl for the detection of *BRAF* V600E mutation at ≥ 5%) (Drugs@FDA: FDA-Approved Drugs^5^). In contrast to the Cobas 4800 test, the THxID test has a high degree of sensitivity for both V600E and V600K. In fact, the Cobas® test was found to detect only 70% of V600K (Anderson et al., 2012). Recently, CE-IVD real-time PCR tests have been developed to detect all *BRAF* V600 mutations with a sensitivity similar or superior to that observed with pyrosequencing. This is the case of Peptide Nucleic Acid (PNA)-mediated PCR clamping, a real-time PCR method which uses PNAs to bind normal DNA sequences, so that only mutant DNA is amplified. Due to its high sensitivity and specificity (99.5 and 100%), PNA-mediated PCR clamping offers a valid alternative to pyrosequencing (Jeong et al., 2012; Bruno et al., 2017). Indeed, PNA clamp real-time PCR detected a 0.5% *BRAF* V600E mutant in the background of the WT with high sensitivity since it is based on the principle that PNA inhibits WT by hybridizing normal sequences, and therefore mutant DNA is preferentially amplified.

Another PCR-based technology is the dPCR that is a cost-effective, sensitive method for detecting mutated DNA in tissue or blood samples. dPCR, in its different formats (chamber dPCR and droplet dPCR), differs to quantitative real-time PCR, since DNA mutants can be quantified here without the need for calibration curves (Day et al., 2013). A drawback of this technology is that it is able to detect single mutations or sets of highly related mutations at the same locus. Malicherova B et al. compared the sensitivity of four *BRAF* V600E detection methods [Cobas® 4800 system based on real-time PCR amplification, Sanger sequencing, allele-specific PCR (AS-PCR), and droplet digital PCR (ddPCR) in FFPE melanoma biopsies from 87 consecutive melanoma patients (stage I-V disease)]. The results indicated good agreement among all four methods about the presence of the *BRAF* V600E mutation reaching a concordance between Cobas® 4800 and ddPCR of 88.9% for *BRAF* and 100% for WT patients. In addition, ddPCR was able to detect the *BRAF* V600E mutation in eight patients that would not have been identified by the others techniques thanks to its detection limit of 0.001% (Malicherova et al., 2018). Similarly, in another recent work, researchers compared ddPCR with Sanger sequencing and pyrosequencing in 40 melanoma FFPE tissues regarding the detection rates of mutations in *BRAF, NRAS*, and *TERT* promoter. The study revealed the ddPCR as the most sensitive method, followed by pyrosequencing and then Sanger sequencing. Concordance between the platforms was high in tumors with high neoplastic cell content, whereas, at low tumor cellularity, the sensitivity offered by ddPCR allowed for the detection of mutations at low frequency abundance (McEvoy et al., 2018). In addition, Lamy PJ et al. analyzed *BRAF* V600E mutation in 47 metastatic melanoma biopsies by dPCR reaching a LOD of 0.0195% mutated allele. The four assessed methods in this study showed a good concordance, especially when samples with high tumor cellularity are analyzed. In particular, *BRAF* V600E mutation was detected in 22 samples (46.8%) by dPCR, in 21 samples (44.7%) by allele-specific amplification, and in 19 samples (40.4%) samples by HRM and pyrosequencing (Lamy et al., 2015). Finally, Bisschop C et al. analyzed 39 FFPE melanoma tissue samples collected by IHC using the anti-BRAF-V600E (VE1) mouse monocolonal antibody (BRAF-VE1 IHC), a V600E-specific ddPCR Test, and the Idylla BRAF- Mutation Test (Idylla). In this study, ddPCR showing a sensitivity of 100% and a specificity of 95% for *BRAF* V600E (Bisschop et al., 2018). Based on these results, ddPCR should be the primary method of detecting and monitoring *BRAF* V600E-mutated cutaneous melanomas as methods of screening thanks to the higher sensitivity and lower LOD of mutant allele. Moreover, the higher sensitivity of this method could allow investigating tumor cell mutation heterogeneity and its changes during tumor progression in a more thorough way than with the other methods.

### HRM Analysis

HRM analysis is a PCR-based method for the identification of *BRAF* mutations. HRM of nucleic acids depends on the ability to record and evaluate fluorescence intensities as a function of the melting temperature of PCR products. The distinctive melting curve can be used to detect DNA sequence variations in the amplicon without the need for any post-PCR processing. The method is easy to use, highly sensitive, specific, low in cost, and yields rapid sample turn-around. Indeed, HRM compared to other methods is an in-tube method in which the analysis is performed immediately after the amplification and is thus particularly suitable to give a quick response to oncologists on *BRAF* mutation status. Moreover, HRM analysis has an detection limit around 5.0% mutated alleles (Carbonell et al., 2011; Ney et al., 2012). Franczak C et al. assessed and compared *BRAF* mutations in 59 FFPE melanoma samples using HRM PCR, real-time Allele-Specific Amplification (RT-ASA) PCR, NGS, IHC, and the diagnostics platform Idylla^TM^. Sensitivity and specificity for HRM were 87.1 and 96.4%, respectively and was the less accurate assay for the detection of *BRAF* mutation on exon 15 (Harlé et al., 2016; Franczak et al., 2017). Marchant J et al. compared the THxID™-BRAF diagnostic test with HRM and Sanger sequencing in 113 melanoma FFPE samples. Although HRM have a relative high sensitivity for mutation detection, it failed to detect three patients with V600 mutation (Marchant et al., 2014). In contrast, Ihle et al. showed no difference in sensitivity between the HRM analysis and Sanger sequencing (98%). All mutations down to 6.6% allele frequency could be detected with 100% specificity (Ihle et al., 2014). In 2014, a meta-analysis of 14 studies involving 1,324 samples in the detecting *BRAF* mutation indicated that the overall values of the sensitivity and specificity of HRM were 0.99 and 0.99, respectively (Chen et al., 2014). Richter et al. carried out a blinded study to evaluate various *BRAF* mutation testing methodologies in FFPE melanoma samples using Sanger sequencing, single-strand conformation analysis (SSCA), HRM and Competitive Allele-Specific TaqMan® PCR (CAST-PCR). Concordance of 100% was observed between the Sanger sequencing, SSCA and HRM techniques (Richter et al., 2013). In general, the real-time PCR-based methods achieved a lower LOD than Sanger sequencing, pyrosequencing and HRM analysis (it may detect <1% of mutated DNA).

## MALDI-TOF MS

MALDI-TOF MS allows multiplexed genotyping and is as a sensitive, reliable, fast, and cost-effective method. The general principle of MALDI-TOF MS is based on amplification of the DNA by PCR, resulting in copies of both mutant and wildtype alleles. Then, primer extension performed using terminator nucleotides A, C, T, and G, each with distinct masses leads to different masses of the amplicons depending on the mutational status that can subsequently be detected by mass spectrometry. Different platforms have been developed by several companies for the simultaneously analysis of various mutations by mass spectrometry, such as MassARRAY® Dx 4 system (Agena Bioscience Inc., San Diego, CA) and Sequenom MassARRAY (Sequenom, San Diego, CA). MassARRAY® system is a non-fluorescent detection platform utilizing mass spectrometry to accurately measure PCR-derived amplicons providing accurate, rapid, and cost-effective analysis (Agena Bioscience Inc., San Diego, CA) with a high level of accuracy and reproducibility. In this context, several commercial panel for somatic mutation profiling using MALDI-TOF MS platforms are available. The iPLEX HS Melanoma panel (Agena Bioscience Inc., San Diego, CA) detects 97 clinically relevant variants in 11 melanoma relevant genes, including *BRAF*, at as low as 1% minor allele frequency from FFPE tissue. Interestingly, Greaves WO et al. compared the sensitivity of MALDI-TOF custom assay (Sequenom MassARRAY; Sequenom, San Diego, CA) with pyrosequencing in the detection of *BRAF* mutations in 145 specimens, including melanomas, finding a concordance between both assays of 99.3% (144/145). Using pyrosequencing as the gold standard, the sensitivity and specificity of the MALDI-TOF assay were 97.6% and 100%, respectively (Greaves et al., 2013). In conclusion, MALDI-TOF MS is a robust approach for the genotyping of known mutations with high sensitivity and specificity reaching a LOD of 1–5% of mutant DNA. Moreover, it is versatile, thanks the capability to create custom assays, and have the advantages to multiplexing, such as NGS. However, it has drawbacks such as expensive instruments consumables and with an elaborate post-PCR processing technique.

## NGS

NGS provides far more genetic information: besides single point mutations, NGS allows detection of additional variant types and simultaneous analysis of multiple genes. The importance of multi-gene analysis in melanoma is expected to increase, as more pharmacologically actionable oncogenic mutations will be discovered, and additional targeted treatments become available. NGS techniques are more sensitive than many real-time PCR assays even when tumor DNA represents <10% of the total DNA. In a study comparing different methods, NGS had 100% specificity and 99% sensitivity, and could be used to study all samples, including those with limited tumor tissue (Ihle et al., 2014). Recently, 21 melanoma samples were analyzed with Ion AmpliSeq™ Cancer Hotspot Panel v2 in order to compare NGS performance with the Sequenom MassARRAY system, Sanger sequencing, and allele-specific real-time PCR. NGS identified two correctly mutation at very low frequency in the *BRAF* gene missed both by mass-spectrometry and Sanger sequencing, confirming the high sensitivity of NGS method (LOD of 5%) (Mancini et al., 2019). On the other hand, NGS demands expertise in data analysis and its application in a clinical context requires an established workflow to obtain reliable sequencing results. Moreover, it requires more time and is more expensive than most of the other techniques. Since there is no targeted drug available for most alterations that can be detected with NGS, a comprehensive screening of multiple genes by NGS nowadays is considered as a research tool more than a technique to be used in everyday practice. However, limiting the analysis to currently actionable genes (i.e. *BRAF, NRAS*, and *KIT*) in one single experiment seems cost and time effective in the diagnostic setting. Unlike the other methods, it also shows the advantage of a quantitative detection of variant allele frequency. In addition to the NGS panels offered through service, the laboratories use commercial panels approved CE-IVD (when available), commercial panels formally aimed only for research use and panel developed by clinical researchers (laboratory developed techniques, LDT). Based on current guidelines, the use of panels no CE-IVD for the diagnostic is allowed where it concerns validated tests for which an internal and external quality assessment laboratory verification phase has been carried out. For the external quality assessment, the European Molecular Genetics Quality Network (EMQN) provide several schemes, such as melanoma scheme to evaluate the capability to assess genotyping and clinical interpretation of *BRAF* gene mutations and NEXTGEN (S) for assessment of genotyping and quality of somatic NGS raw data (http://www.emqn.org/emqn/Home). The implementation of an NGS test in a diagnostic laboratory is an extremely complex process that must be documented in a timely manner. Validation of a new diagnostic test involves definition of the performance specifications that it must meet and the demonstration that its performance has been achieved in terms of accuracy, limits and accuracy of the results (Jennings et al., 2017). For this purpose, diagnostic specimens or commercially available reference standard controls containing the same mutations of interest should be used for validation. It is also imperative to include a broad spectrum of allele frequencies of the variants investigated to establish the detection limits for the various types of mutation. Moreover, the validation must identify the limitations of the technique including the amount of DNA needed and the minimal tumor cells content in order to guarantee adequate analysis result. For molecular laboratories without these capacities, the use of pre-tested and validated panels available on the market may be the best option, given the complexity of validating an LDT test. However, even if commercial panels are used, both IVD and research, it is essential that the laboratory validated it before its implementation in clinical diagnostics. Actually, several companies developed CE-IVD NGS panels, but, to date, only Sentosa SQ Melanoma Panel (Vela Diagnostics) is specific for melanoma; it is able to detect 127 hot spot mutations and sequence variants in 10 melanoma genes with a mutation detection sensitivity of 5%. Moreover, the only melanoma NGS panel approved by the FDA is the FoundationOne CDx™ (F1CDx™) that it is able to detect substitutions, insertion and deletion alterations (indels), and Copy Number Alterations (CNAs) in 324 genes and select gene rearrangements, as well as genomic signatures, including microsatellite instability and TMB using DNA isolated from FFPE tumor tissue. However, the application of FoundationOne CDx™ is not recommended in routinely cutaneous melanoma diagnostic test. In summary, in order to perform the full characterization required in advanced cutaneous melanoma diagnostic setting, the employment of NGS panel resulted faster and required less DNA compared to other diagnostic methods that analyze single mutation or region. These aspects are fundamental in terms of working days and to spare sample for confirmatory tests. Currently, NGS is already used in clinical practice in several centers (Reiman et al., 2017; Mancini et al., 2019; Park et al., 2019). In the future, it is expected that NGS will be routine in molecular diagnostics, in view of the high sensitivity and multiplexing options that allow the molecular profiling of each tumor sample (Spagnolo et al., 2015). In a study performed on 100 primary melanomas matched with 25 metastatic tumors aimed to identify the best combination of methods for detecting *BRAF* mutations (among PNA-clamping real-time PCR, IHC, and Sanger sequencing), a *BRAF* mutation frequency of 62%, based on the combination of at least two techniques was obtained (Bruno et al., 2017). Concordance between mutation status in primary and metastatic tumor was good but not complete (67%), when agreement of at least two techniques were considered. NGS was used to quantify the threshold of detected mutant alleles in discordant samples. Combining different methods excludes that the observed heterogeneity is technique-based. Therefore, an algorithm for *BRAF* mutation testing based on agreement between IHC and PNA was proposed; a third molecular method could be added in case of discordance of the results. Testing the primary tumor when the metastatic sample is unavailable is a good option if at least two methods of detection are used; however, the presence of intertumoral heterogeneity or the occurrence of additional primaries should be carefully considered (Bruno et al., 2017).

### Diagnostic Algorithm

Overall, in the daily diagnostics and clinical practice, a sequential analysis of two methods, with initial detection of V600E-positive cases by IHC together with a molecular mutation testing technique, such as Sanger sequencing, followed by pyrosequencing or real-time PCR-based, or NGS, can be suggested. In fact, the use of IHC alone carries a significant risk of false negative results and should not be considered unless the sample DNA yield is no sufficient for molecular analysis. On the other hand, a positive result from a sensitive molecular technique may reflect a minor *BRAF*-mutated subclone in a predominantly wild-type tumor, and this is thus not clinically relevant in terms of response to targeted therapy. As described above, an NGS approach to the detection of *BRAF, NRAS*, and *KIT* mutations in one single step can be considered in clinical practice, with the advantage of high sensitivity and quantitative information in terms of variant allele frequency and a lower cost compared to the single analysis of the three genes.

## Emerging Techniques: Liquid Biopsy

**Figure 2:**
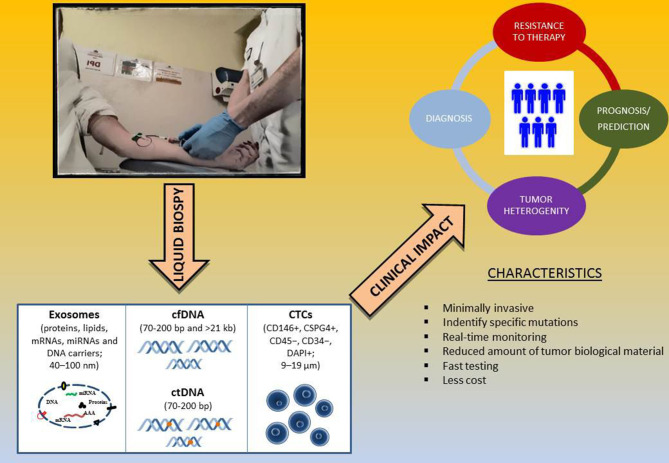
Liquid biopsy as surrogate biomarker in melanoma. Abbreviations: cfDNA, cell free DNA; ctDNA, circulating tumor DNA; CTCs, circulating tumor cells.

Liquid biopsy is a non-invasive diagnostic technique for the evaluation of tumor genetic status based on the analysis of circulating free DNA (cfDNA) from different body fluids, such as plasma and serum. Since blood samples are easily obtainable, plasma or serum biopsy has long been considered as a promising non-invasive method to be integrated into traditional biopsy techniques for molecular analysis. One way in which tumors provide information in the form of biomarkers, such as circulating tumor DNA (ctDNA), is through tumor cell necrosis with subsequent release of dead cells or cellular debris. Afterwards, phagocytes incorporate these cells processing and then release the ctDNA in blood in the form of small fragments. ctDNA is the cfDNA that is shed from tumor cells, and it can be analyzed for the same genetic alterations found in the tumor (Lipson et al., 2014; Knol et al., 2016; Gangadhar et al., 2018; Long-Mira et al., 2018). In addition to ctDNA, Circulating tumor Cells (CTCs) or exosomes can provide tumor DNA for liquid biopsy. Besides ctDNA, CTCs are intact tumor cells released into the bloodstream that can be used as a source to extract tumor DNA (Zhang et al., 2016a). Finally, exosomes are extracellular membrane vesicles, with a size between 40 and 100nm, that are released by most cells, including cancer cells, and they therefore can be used in liquid biopsy as a source of DNA, RNA, and tumor proteins (Vanni et al., 2017; Figure 2).

### cfDNA/ctDNA

cfDNA in blood was first discovered in 1948 and has emerged as a promising diagnostic tool for cancer patients (Diaz and Bardelli, 2014). The total amount of cfDNA in the plasma and serum of cancer patients varies from patient to patient but cancer patients have higher plasma and serum cfDNA levels than patients without cancer (Leon et al., 1977; Fournié et al., 1995). Indeed, cfDNA concentration in blood varies significantly; it ranges between 0–5 and >1000 ng/ml in patients with cancer and between 0 and 100 ng/ml in healthy subjects (Schwarzenbach et al., 2011). In general, several studies have shown that cfDNA is present in small quantities also in the blood of healthy individuals and increases in patients suffering from a series of clinical disorders such as cancer, stroke, trauma, myocardial infarction, autoimmune diseases, and complications associated with pregnancy (Swarup and Moganty, 2007). In melanoma cancer patients, plasma cfDNA levels are higher in advanced patients with a median of 135 pg/μl ranging from 17 pg/μl to 1.125 pg/ml of plasma compared to early stage (Valpione et al., 2018). There are several hypotheses regarding the release of cfDNA in the circulatory stream, but the one most accepted by the scientific community consists in the necrosis and apoptosis of the tumor cells or through their active release (Stroun et al., 2001). According to this hypothesis, apoptotic and necrotic cancer cells and DNA strands, which are not phagocytized, enter in the bloodstream as cfDNA (Jahr et al., 2001). The estimated size of cfDNA varies from ~40–200 bp, with a peak at about 166 bp, which are characteristic of the apoptotic process (Snyder et al., 2016; Zhang et al., 2016b; Mouliere et al., 2018). However, individual cfDNAs might carry thousands of base pairs (>20–30 kb) as result from necrotic cell death (Thierry et al., 2016). In the cancer context, liquid biopsy in metastatic melanoma has emerged as a complementary tool to tumor biopsies for detection of actionable alterations. Indeed, it enables non-invasive and quantitative characterization of the whole tumor genome, identification of tumor heterogeneity, and clonal evolution during treatment and toward disease progression in cancer patients, including melanoma (Ascierto et al., 2013; Bettegowda et al., 2014; Lebofsky et al., 2015; Sanmamed et al., 2015; Santiago-Walker et al., 2016). As it is known, a requirement for administration of BRAFi/MEKi is the identification of a *BRAF* mutation in specific melanoma samples, which, however, may not represent the current somatic mutation status or tumor heterogeneity. ctDNA analysis allowing for real-time comprehensive mutation assessment of all tumor sites within a patient may overcome this limitation. Beyond that, the minimally invasive nature of ctDNA sample acquisition enables routine monitoring of response and resistance to targeted therapy. Nevertheless, the disadvantages of using ctDNA for molecular investigations are the follows: (i) very low ctDNA concentrations that are often not sufficient to carry out molecular investigations; (ii) ctDNA which represents between 0.01 and 10% of the total cfDNA and therefore the need for highly sensitive technologies for the evaluation of the presence of genetic alterations of interest; and (iii) the very fragmented nature of the ctDNA, which requires tests for the detection of genetic alterations of interest suitable for the analysis of portions of DNA of about 100–200 bp.

Chiefly, its highly fragmented nature (Diehl et al., 2005, 2008; Fleischhacker et al., 2011) and the minor fraction of ctDNA with a variable contribution of 0.01 to 10% of total cfDNA (Diehl et al., 2005; Dawson et al., 2013) are of high importance with respect to the desired sensitivity of the selected detection method and the preanalytical sample handling. Indeed, the most common mistakes include the selection of the use of inappropriate blood collection tube (e.g., hemolysis or insufficient volume), wrong sample storage and transportation, and inappropriate DNA extraction and mutation detection tests. However, to date there are no integrated, multicenter-tested workflows available covering the requirements for the use of liquid biopsy in the clinical setting. Indeed, currently there is only one FDA-approved ctDNA based mutation test but only for the detection of *EGFR* mutations in NSCLC patients: the Cobas EGFR Mutation Test v2 (Cobas EGFR Mutation Test v2. 2016. Available online at: http://www.fda.gov/Drugs/InformationOnDrugs/ApprovedDrugs/ucm504540.htm).

Although many different liquid biopsy technologies have been recently developed, there is still a lack of established workflows from the sample to clinically meaningful data. In addition, many standard molecular biology techniques for the detection of potentially targeting melanoma mutations are not suitable for the analysis of ctDNA due to their low sensitivity. In fact, since ctDNA often represents a small percentage of the total cfDNA, somatic mutations from the tumor can be present in extremely low allele fractions (up to 0.01%). For this reason, highly sensitive methodologies or modifications of pre-existing technologies have been developed in order to detect low-frequency mutations in cfDNA of melanoma patients, such as Allele-specific amplification Refractory Mutation System PCR (ARMS), Bead Emulsification Amplification and Magnetics (BEAMing) technology, Allele-Specific PCR (AS-PCR), PNA-PCR clamping technique, ddPCR, and NGS (Crowley et al., 2013; Volik et al., 2016; Busser et al., 2017; Table 2). However, in metastatic melanoma, there is usually enough tumor tissue available for genetic analyses. Therefore, the mutation testing in the liquid biopsy of melanoma patients is not to be a surrogate of solid biopsy and it should only be limited in case there is not enough material or in order to offer additional clinically relevant information, such as clonality and tumor heterogeneity. It is notable that the liquid biopsy, and in particular cfDNA and CTCs, are not only important for the detection of clinically relevant mutations in melanoma but also for its prognostic and predictive value for patient outcome and response to therapy (Busser et al., 2017; Gaiser et al., 2018).

**Table 2:** Overview of techniques used for the detection of *BRAF* mutation in cfDNA from melanoma patients.

**Method**	**Gene** **(mutation)**	**Sensitivity** **(% of mutated copies)**	**References**
ddPCR	BRAF V600E	0.001%	Burjanivova et al., 2019
ddPCR	TERT promoter	0.031–0.063%	Corless et al., 2019
ddPCR	BRAF V600E	0.001%	Malicherova et al., 2018.
ddPCR	BRAF V600E		Tang et al., 2018
ddPCR	TERT promoter	0.17%	McEvoy et al., 2017
ddPCR	BRAF V600E, BRAF V600K, NRAS Q81K, NRAS Q61R	0.1%	Gray et al., 2015
ddPCR	BRAF V600E, BRAF V600K, NRAS Q81K	0.01	Chang-Hao Tsao et al., 2015
ddPCR	BRAF V600E	0.001	Sanmamed et al., 2015
MassArray System	13 genes		Gray et al., 2019
BEAMing technology	BRAF V600E, BRAF V600K	0.01%	Santiago-Walker et al., 2016
BEAMing technology	BRAF V600E, NRAS Q81K, NRAS Q61R	<0.01	Lipson et al., 2014
BEAMing technology	BRAF V600E, BRAF V600K	0.01	(Ascierto et al., 2013; 2118436)
BEAMing technology	BRAF V600E, BRAF V600K, NRAS Q61K, NRAS Q61R, NRAS Q61L, NRAS Q61H	0.03%	Rowe et al., 2018
Quantitative real-time clamp reverse transcription PCR	BRAF V600E	0.001	Shinozaki et al., 2007
CastPCR	BRAF V600E	0.5	Ashida et al., 2016
AS-real-time PCR	BRAF V600E	0.3%	Pinzani et al., 2010
AS-PCR or ARMS	BRAF V600E, BRAF V600K, BRAF V600D	0.1%	Board et al., 2009
AS-PCR or ARMS	BRAF V600E	0.3%	Cao et al., 2015
AS-PCR or ARMS	BRAF V600E	0.25%	Daniotti et al., 2007
AS-PCR or ARMS	BRAF V600E	2%	Aung et al., 2014
ARMS	BRAF V600E, BRAF V600K, BRAF V600R, BRAF V600D	1.82–4.85%	Knol et al., 2016
AS-PCR	BRAF V600E, BRAF V600K, BRAF V600R, BRAF V600D	0.01%	Schreuer et al., 2016
Mutant-specific PCR	BRAF V600E	0.01%	Yancovitz et al., 2012
Real-time PCR + restriction enzyme digestion	BRAF V600E	0.1%	Panka et al., 2010
Biochip assay (Nested LNA-Clamp PCR)	BRAF V600E, BRAF V600M, BRAF V600K, BRAF V600R, BRAF V600D		Emelyanova et al., 2018
NGS	Exome		Girotti et al., 2015
NGS	Whole genome		Cutts et al., 2017

### CTCs and Exosomes

The CTCs evaluation for the identification of melanoma actionable mutations is limited compared to ctDNA assessment since the use of either CTCs marker or size-based enrichment methods leads to the loss of surface marker negative or small CTCs, respectively. Moreover, the typically only 1–10 CTCs can be found in 4 ml of blood of metastatic melanoma patients, and, for this reason, CTC enrichment methods and a highly sensitivity method are mandatory in order to identify melanoma mutations (Freeman et al., 2012). To date, therefore, the clinical utility of melanoma CTCs is still unclear due to rarity and heterogeneity and a lack of a standardized isolation. Sakaizawa K et al. have analyzed CTC isolated from blood of 11 melanoma patients using immunomagnetic beads coated with HMW-MAA-specific antibodies followed by immunohistochemical laser dissection techniques in order to genotypes *BRAF* and *KIT* genes by PCR amplifications. This study highlighted that the genotypes of the CTC differed from those of the primary tumors, and the metastatic lesions suggest clonal heterogeneity. The success rate of PCR amplification for *BRAF* evaluation and *KIT* from single CTC ranged from 20 to 100% and from 0 to 50%, respectively (Sakaizawa et al., 2012). In a more recent study, researchers used immunomagnetic beads for CTCs enrichment from metastatic melanoma patients blood with BRAF mutated tumors (Reid et al., 2015). The DNA extracted from CTCs has been subjected to Wool Genome Amplification (WGA) and tested for *BRAF* V600E or V600K mutations by ddPCRs. The study demonstrated that WGA combined with ddPCR allows the detection of cancer mutations in CTCs after partial isolation by enrichment since *BRAF* V600E mutations was found in all patients at a fractional abundance ≥ 0.0005% (Reid et al., 2015).

Kiniwa Y et al. used a high-density dielectrophoretic microwell array following by single-cell sequencing in 33 CTCs to reveal *BRAF* status from a patient with advanced melanoma revealing an heterogeneous *BRAF* status in CTCs (Kiniwa et al., 2018). On the other hand, Hofman V et al. has combined ISET and IHC using the VE1 antibody to investigate the presence of *BRAF* V600E in CTC isolated in 87/98 (89%) melanoma patients. In particular, of 87 patients, 54 (62%) demonstrated positive immunostaining on ISET filters, and, among 46 (85%) patients with *BRAF* mutation, the V600E mutation was also identified in tissue specimen by pyrosequencing. In contrast, 8/54 (15%) patients with positive VE1-immunostained CTCs lacked *BRAF* V600E in tumor tissues (Hofman et al., 2013).

Finally, very few studies have been carried out on the *BRAF* mutation status in DNA inside exosomes in melanoma (García-Silva et al., 2019). It is well-known that exosomes, besides their specific surface proteins, also carry a select set of functional circulating nucleic acids such as mRNAs, miRNAs, LncRNAs, and DNA (Schwarzenbach et al., 2011). Very recently, Garcia et al. found that the *BRAF* V600E mutation can be detected in exudative seroma (ES)-derived extracellular vesicles by quantitative PCR (LOD of 0.01%) and also correlated with risk of relapse. The researchers pointed out as the detection of *BRAF* mutation in ES vesicles obtained through lymphatic drainage may be a novel parameter to identify melanoma patients at risk of relapse probably due to the presence of residual disease (García-Silva et al., 2019).

In conclusion, despite the high potential of liquid biopsy, its systematic application in real practice is still limited, especially in the detections of relevant mutations, due to many hindrances, such as unsatisfactory specificity and sensitivity, lack of standardization, and elevated economic and human resource costs; it thus still offers many challenges. Last but not least, the onset of drug resistance mechanisms could be identified in melanoma patients using the liquid biopsy (Figure 2).

## Conclusion and Perspectives

In summary, with the introduction of effective targeted therapy for the treatment of *BRAF*-mutant melanoma, which account for about 50% of all melanomas, it is now mandatory to assess the molecular status of all stage IV patients and, in light of the approval of BRAF and MEK inhibitors in the adjuvant setting, of patients with high-risk, resected stage III melanoma. Therefore, molecular testing for *BRAF* mutations is a priority in determining the course of therapy. In this view, several different techniques for *BRAF* testing are currently developed and used by various laboratories. The choice of methods employed by the center should take into account the specific sensitivity, reproducibility, accuracy, running time, and analysis cost of the assay. Moreover, the choice and use of techniques able to quantify the mutant allele frequency of *BRAF* gene is important since it is reported to influence the clinical efficacy of the BRAF/MEK inhibitors treatments. So, quantitative analysis of the *BRAF* gene could be useful to select the melanoma patients who are most likely to benefit from target therapy (Stagni et al., 2018). In addition, intertumoral and intratumoral heterogeneity could lead to misinterpretation of *BRAF* mutational status; this is especially important if testing is performed on primary specimens, or micromestasis, when abundant metastatic lesions are unavailable. In this context, the characterization of molecular heterogeneity in advanced melanoma patients could be overcome by the analysis of liquid biopsy that represent an efficient non-invasive tool to overcome the problem. Potential of circulating biomarkers in liquid biopsy diagnostics holds promise as a tool for diagnosis and treatment of melanoma, including early diagnosis screening, tumor heterogeneity, drug resistance, and establishment of targets. Several efforts are being made to standardize extraction and molecular analysis technologies and protocols in order to implement the liquid biopsy assays in cancer field hoping it could be replacing tissue biopsy in the near future. In fact, the need to obtain recurrent molecular assessments will become increasingly greater in order to offer the most suitable therapy for the patient from time to time. Moreover, liquid biopsy may be more representative of patient's whole disease than an isolated portion of tumor tissue, and this is particularly important due to tumor heterogeneity. The opportunity to obtain these assessments without recurring to invasive procedures and, above all, being able to evaluate patients with surgically inaccessible lesions will be a milestone in the treatment of melanoma.

## Author Contributions

IV and ET contributed equally in conceiving the review focus, conducting the literature review, summarizing the manuscript, writing the first draft, and finalizing the manuscript. WB and PG designed and directed the review. FS, VA, WB, and PG revised and made corrections to the manuscript. All authors have read and agreed to the final version of the manuscript.

## Conflict of Interest

The authors declare that the research was conducted in the absence of any commercial or financial relationships that could be construed as a potential conflict of interest.

## Footnotes

^1^*Global Cancer Observatory*. Available Online at: http://gco.iarc.fr/ (accessed November 23, 2019).

^2^AIOM, *AIRTUM. I numeri del cancro in Italia 2019*. Available online at: https://www.aiom.it/wp-content/uploads/2019/09/2019_Numeri_Cancro-operatori-web.pdf (accessed January 10, 2020).

^3^*NCCN Clinical Practice Guidelines in Oncology*. Available Onlineat: https://www.nccn.org/professionals/physician_gls/default.aspx (accessed 27 January 2020).

^4^*Biomérieux Corporate Website*. Available online at: https://www.biomerieux.com/en (accessed January 27, 2020).

^5^*Drugs@FDA: FDA-Approved Drugs*. Available Online at: https://www.accessdata.fda.gov/scripts/cder/daf/ (accessed January 27, 2020).

## References

[B1] Akbani R. Akdemir K. C. Aksoy B. A. Albert M. Ally A. Amin S. B. (2015). Genomic classification of cutaneous melanoma. Cell 161, 1681–1696. 10.1016/j.cell.2015.05.044. 26091043 PMC4580370

[B2] Alexandrov L. B. Nik-Zainal S. Wedge D. C. Aparicio S. A. J. R. Behjati S. Biankin A. V.. (2013). Signatures of mutational processes in human cancer. Nature 500, 415–421. 10.1038/nature12477 23945592 PMC3776390

[B3] Anderson S. Bloom K. J. Vallera D. U. Rueschoff J. Meldrum C. Schilling R.. (2012). Multisite analytic performance studies of a real-time polymerase chain reaction assay for the detection of BRAF V600E mutations in formalin-fixed, paraffin-embedded tissue specimens of malignant melanoma. Arch. Pathol. Lab. Med. 136, 1385–1391. 10.5858/arpa.2011-0505-OA 22332713

[B4] Ascierto P. Minor D. Ribas A. Lebbé C. O'Hagan A. Arya N.. (2013). Phase II trial (BREAK-2) of the BRAF inhibitor dabrafenib (GSK2118436) in patients with metastatic melanoma. J. Clin. Oncol. 31, 3205–3211. 10.1200/JCO.2013.49.8691 23918947

[B5] Ashida A. Sakaizawa K. Mikoshiba A. Uhara H. Okuyama R. (2016). Quantitative analysis of the BRAF V600E mutation in circulating tumor-derived DNA in melanoma patients using competitive allele-specific TaqMan PCR. Int. J. Clin. Oncol. 21, 981–988. 10.1007/s10147-016-0976-y 27041702

[B6] Aung K. Donald E. Ellison G. Bujac S. Fletcher L. Cantarini M.. (2014). Analytical validation of BRAF mutation testing from circulating free DNA using the amplification refractory mutation testing system. J. Mol. Diagn. 16, 343–349. 10.1016/j.jmoldx.2013.12.004 24631158

[B7] Barbour A. Tang Y. Armour N. Dutton-Regester K. Krause L. Loffler K.. (2014). BRAF mutation status is an independent prognostic factor for resected stage IIIB and IIIC melanoma: implications for melanoma staging and adjuvant therapy. Eur. J. Cancer 50, 2668–2676. 10.1016/j.ejca.2014.06.009 25070294

[B8] Berger M. F. Hodis E. Heffernan T. P. Deribe Y. L. Lawrence M. S. Protopopov A.. (2012). Melanoma genome sequencing reveals frequent PREX2 mutations. Nature 485, 502–506. 10.1038/nature11071 22622578 PMC3367798

[B9] Bettegowda C. Sausen M. Leary R. J. Kinde I. Wang Y. Agrawal N.. (2014). Detection of circulating tumor DNA in early- and late-stage human malignancies. Sci. Transl. Med. 6:224ra24. 10.1126/scitranslmed.3007094 24553385 PMC4017867

[B10] Bisschop C. Ter Elst A. Bosman L. J. Platteel I. Jalving M. van den Berg A.. (2018). Rapid BRAF mutation tests in patients with advanced melanoma: comparison of immunohistochemistry, droplet digital PCR, and the idylla mutation platform. Melanoma Res. 28, 96–104. 10.1097/CMR.0000000000000421 29232304 PMC5844592

[B11] Board R. E. Ellison G. Orr M. C. M. Kemsley K. R. McWalter G. Blockley L. Y.. (2009). Detection of BRAF mutations in the tumor and serum of patients enrolled in the AZD6244 (ARRY-142886) advanced melanoma phase II study. Br. J. Cancer 101, 1724–1730. 10.1038/sj.bjc.6605371 19861964 PMC2778539

[B12] Bowyer S. Rao A. Lyle M. Sandhu S. Long G. Mcarthur G.. (2014). Activity of trametinib in K601E and L597Q BRAF mutation-positive metastatic melanoma. Melanoma Res. 24, 504–508. 10.1097/CMR.0000000000000099 24933606

[B13] Bruno W. Martinuzzi C. Andreotti V. Pastorino L. Spagnolo F. Dalmasso B.. (2017). Heterogeneity and frequency of BRAF mutations in primary melanoma: comparison between molecular methods and immunohistochemistry. Oncotarget 8, 8069–8082. 10.18632/oncotarget.14094 28039443 PMC5352383

[B14] Burjanivova T. Malicherova B. Grendar M. Minarikova E. Dusenka R. Vanova B.. (2019). Detection of BRAFV600E mutation in melanoma patients by digital PCR of circulating DNA. Genet. Test. Mol. Biomarkers 23, 241–245. 10.1089/gtmb.2018.0193 30676087

[B15] Busam K. J. Hedvat C. Pulitzer M. von Deimling A. Jungbluth A. A. (2013). Immunohistochemical analysis of BRAF(V600E) expression of primary and metastatic melanoma and comparison with mutation status and melanocyte differentiation antigens of metastatic lesions. Am. J. Surg. Pathol. 37:413e420. 10.1097/PAS.0b013e318271249e 23211290

[B16] Busser B. Lupo J. Sancey L. Mouret S. Faure P. Plumas J.. (2017). Plasma circulating tumor DNA levels for the monitoring of melanoma patients: landscape of available technologies and clinical applications. Biomed Res. Int. 2017:5986129. 10.1155/2017/5986129 28484715 PMC5397613

[B17] Cao M. Mayo C. Molina-Vila M. De Mattos-Arruda L. Muñoz-Couselo E. Manzano J.. (2015). BRAF mutation analysis in circulating free tumor DNA of melanoma patients treated with BRAF inhibitors. Melanoma Res. 25, 486–495. 10.1097/CMR.0000000000000187 26366702

[B18] Capper D. Berghoff A. S. Magerle M. Ilhan A. Wöhrer A. Hackl M.. (2012). Immunohistochemical testing of BRAF V600E status in 1,120 tumor tissue samples of patients with brain metastases. Acta Neuropathol. 123, 223–233. 10.1007/s00401-011-0887-y 22012135

[B19] Carbonell P. Turpin M. C. Torres-Moreno D. Molina-Martínez I. García-Solano J. Perez-Guillermo M.. (2011). Comparison of allelic discrimination by dHPLC, HRM, and TaqMan in the detection of BRAF mutation V600E. J. Mol. Diagn. 13, 467–473. 10.1016/j.jmoldx.2011.03.009 21708284 PMC3157612

[B20] Casula M. Colombino M. Manca A. Caracò C. Botti G. Ascierto P. A.. (2016). Low levels of genetic heterogeneity in matched lymph node metastases from patients with melanoma. J. Invest. Dermatol. 136, 1917–1920. 10.1016/j.jid.2016.05.103 27265006

[B21] Chang-Hao Tsao S. Weiss J. Hudson C. Christophi C. Cebon J. Behren A.. (2015). Monitoring response to therapy in melanoma by quantifying circulating tumor DNA with droplet digital PCR for BRAF and NRAS mutations. Sci. Rep. 5:11198. 10.1038/srep11198 26095797 PMC4476039

[B22] Chen D. Wang Y.-Y. Chuai Z.-R. Huang J.-F. Wang Y.-X. Liu K.. (2014). High-resolution melting analysis for accurate detection of BRAF mutations: a systematic review and meta-analysis. Sci. Rep. 4:4168. 10.1038/srep04168 24566771 PMC3933866

[B23] Colomba E. Hélias-Rodzewicz Z. Von Deimling A. Marin C. Terrones N. Pechaud D.. (2013). Detection of BRAF p.V600E mutations in melanomas: comparison of four methods argues for sequential use of immunohistochemistry and pyrosequencing. J. Mol. Diagn. 15, 94–100. 10.1016/j.jmoldx.2012.09.001 23159108

[B24] Colombino M. Capone M. Lissia A. Cossu A. Rubino C. De Giorgi V.. (2012). BRAF/NRAS mutation frequencies among primary tumors and metastases in patients with melanoma. J. Clin. Oncol. 30, 2522–2529. 10.1200/JCO.2011.41.2452 22614978

[B25] Corless B. C. Chang G. A. Cooper S. Syeda M. M. Shao Y. Osman I.. (2019). Development of novel mutation-specific droplet digital PCR assays detecting TERT promoter mutations in tumor and plasma samples. J. Mol. Diagn. 21, 274–285. 10.1016/j.jmoldx.2018.09.003 30827467 PMC6419583

[B26] Cormican D. Kennedy C. Murphy S. Werner R. Power D. G. Heffron C. C. B. B. (2019). High concordance of BRAF mutational status in matched primary and metastatic melanoma. J. Cutan. Pathol. 46, 117–122. 10.1111/cup.13393 30430609

[B27] Cristescu R. Mogg R. Ayers M. Albright A. Murphy E. Yearley J.. (2018). Pan-tumor genomic biomarkers for PD-1 checkpoint blockade-based immunotherapy. Science 362:eaar3593. 10.1126/science.aar3593 30309915 PMC6718162

[B28] Crowley E. Di Nicolantonio F. Loupakis F. Bardelli A. (2013). Liquid biopsy: monitoring cancer-genetics in the blood. Nat. Rev. Clin. Oncol. 10, 472–484. 10.1038/nrclinonc.2013.110 23836314

[B29] Curtin J. A. Fridlyand J. Kageshita T. Patel H. N. Busam K. J. Kutzner H.. (2005). Distinct sets of genetic alterations in melanoma. N. Engl. J. Med. 353, 2135–2147. 10.1056/NEJMoa050092 16291983

[B30] Cutts A. Venn O. Dilthey A. Gupta A. Vavoulis D. Dreau H.. (2017). Characterisation of the changing genomic landscape of metastatic melanoma using cell free DNA. NPJ Genom. Med. 2, 1–8. 10.1038/s41525-017-0030-7 29075515 PMC5654504

[B31] Dahlman K. B. Xia J. Hutchinson K. Ng C. Hucks D. Jia P.. (2012). BRAF(L597) mutations in melanoma are associated with sensitivity to MEK inhibitors. Cancer Discov. 2, 791–797. 10.1158/2159-8290.CD-12-0097 22798288 PMC3449158

[B32] Daniotti M. Vallacchi V. Rivoltini L. Patuzzo R. Santinami M. Arienti F.. (2007). Detection of mutated BRAFV600E variant in circulating DNA of stage III-IV melanoma patients. Int. J Cancer 120, 2439–2444. 10.1002/ijc.22598 17315191

[B33] Dankner M. Lajoie M. Moldoveanu D. Nguyen T.-T. Savage P. Rajkumar S.. (2018). Dual MAPK inhibition is an effective therapeutic strategy for a subset of class II BRAF mutant melanomas. Clin. Cancer Res. 24, 6483–6494. 10.1158/1078-0432.CCR-17-3384 29903896

[B34] Dawson S.-J. Tsui D. W. Y. Murtaza M. Biggs H. Rueda O. M. Chin S.-F.. (2013). Analysis of circulating tumor DNA to monitor metastatic breast cancer. N. Eng. J. Med. 368, 1199–1209. 10.1056/NEJMoa1213261 23484797

[B35] Day E. Dear P. H. McCaughan F. (2013). Digital PCR strategies in the development and analysis of molecular biomarkers for personalized medicine. Methods 59, 101–107. 10.1016/j.ymeth.2012.08.001 22926236

[B36] Dhillon A. S. Hagan S. Rath O. Kolch W. (2007). MAP kinase signalling pathways in cancer. Oncogene 26, 3279–3290. 10.1038/sj.onc.1210421 17496922

[B37] Diaz L. A. Bardelli A. (2014). Liquid biopsies: genotyping circulating tumor DNA. J. Clin. Oncol. 32, 579–586. 10.1200/JCO.2012.45.2011 24449238 PMC4820760

[B38] Diehl F. Li M. Dressman D. He Y. Shen D. Szabo S.. (2005). Detection and quantification of mutations in the plasma of patients with colorectal tumors. Proc. Natl. Acad. Sci. U.S.A. 102, 16368–16373. 10.1073/pnas.0507904102 16258065 PMC1283450

[B39] Diehl F. Schmidt K. Choti M. A. Romans K. Goodman S. Li M.. (2008). Circulating mutant DNA to assess tumor dynamics. Nat. Med. 14, 985–990. 10.1038/nm.1789 18670422 PMC2820391

[B40] Dvorak K. Aggeler B. Palting J. McKelvie P. Ruszkiewicz A. Waring P. (2014). Immunohistochemistry with the anti-BRAF V600E (VE1) antibody: impact of pre-analytical conditions and concordance with DNA sequencing in colorectal and papillary thyroid carcinoma. Pathology 46, 509–517. 10.1097/PAT.0000000000000119 25014730 PMC4233678

[B41] Emelyanova M. Telysheva E. Orlova K. Abramov I. Snigiryova G. Ryabaya O. (2018). 5PCell-free circulating BRAF V600 mutations analysis by biochip-based assay and droplet digital PCR in melanoma patients. Ann. Oncol 29, mdy316–004. 10.1093/annonc/mdy316.004

[B42] Fisher K. E. Cohen C. Siddiqui M. T. Palma J. F. Lipford E. H. Longshore J. W. (2014). Accurate detection of BRAF p.V600E mutations in challenging melanoma specimens requires stringent immunohistochemistry scoring criteria or sensitive molecular assays. Hum. Pathol. 45, 2281–2293. 10.1016/j.humpath.2014.07.014 25228337

[B43] Flaherty K. T. Hodi F. S. Fisher D. E. (2012). From genes to drugs: targeted strategies for melanoma. Nat. Rev. Cancer 12, 349–361. 10.1038/nrc3218 22475929

[B44] Fleischhacker M. Schmidt B. Weickmann S. Fersching D. M. I. Leszinski G. S. Siegele B.. (2011). Methods for isolation of cell-free plasma DNA strongly affect DNA yield. Clin. Chim. Acta 412, 2085–2088. 10.1016/j.cca.2011.07.011 21861994

[B45] Fournié G. J. Courtin J. P. Laval F. Chalé J. J. Pourrat J. P. Pujazon M. C.. (1995). Plasma DNA as a marker of cancerous cell death. Investigations in patients suffering from lung cancer and in nude mice bearing human tumors. Cancer Lett. 91, 221–227. 10.1016/0304-3835(95)03742-F 7767913

[B46] Franczak C. Salleron J. Dubois C. Filhine-Trésarrieu P. Leroux A. Merlin J.-L. (2017). Comparison of five different assays for the detection of BRAF mutations in formalin-fixed paraffin embedded tissues of patients with metastatic melanoma. Mol. Diagn. Ther. 21, 209–216. 10.1007/s40291-017-0258-z 28130756

[B47] Freeman J. B. Gray E. S. Millward M. Pearce R. Ziman M. (2012). Evaluation of a multi-marker immunomagnetic enrichment assay for the quantification of circulating melanoma cells. J. Transl. Med 10:192. 10.1186/1479-5876-10-192 22978632 PMC3480925

[B48] Furney S. J. Turajlic S. Stamp G. Thomas J. M. Hayes A. Strauss D.. (2014). The mutational burden of acral melanoma revealed by whole-genome sequencing and comparative analysis. Pigment Cell Melanoma Res. 27, 835–838. 10.1111/pcmr.12279 24913711

[B49] Gaestel M. (2006). MAPKAP kinases - MKs - two's company, three's a crowd. Nat. Rev. Mol. Cell Biol. 7, 120–130. 10.1038/nrm1834 16421520

[B50] Gaiser M. R. von Bubnoff N. Gebhardt C. Utikal J. S. (2018). Liquid biopsy to monitor melanoma patients. J. Dtsch. Dermatol. Ges. 16, 405–414. 10.1111/ddg.13461 29512873

[B51] Gangadhar T. C. Savitch S. L. Yee S. S. Xu W. Huang A. C. Harmon S.. (2018). Feasibility of monitoring advanced melanoma patients using cell-free DNA from plasma. Pigment Cell Melanoma Res. 31, 73–81. 10.1111/pcmr.12623 28786531 PMC5742050

[B52] García-Silva S. Benito-Martin A. Sánchez-Redondo S. Hernández Barranco A. Ximénez-Embún P. Nogués L.. (2019). Correction: use of extracellular vesicles from lymphatic drainage as surrogate markers of melanoma progression and BRAF V600E mutation. J. Exp. Med. 216:1230. 10.1084/jem.2018152204162019c 31010898 PMC6504212

[B53] Girotti M. Gremel G. Lee R. Galvani E. Rothwell D. Viros A.. (2015). Application of sequencing, liquid biopsies, and patient-derived xenografts for personalized medicine in melanoma. Cancer Discov. 6, 238–299. 10.1158/2159-8290.CD-15-1336 26715644

[B54] Goodman A. M. Kato S. Bazhenova L. Patel S. P. Frampton G. M. Miller V.. (2017). Tumor mutational burden as an independent predictor of response to immunotherapy in diverse cancers. Mol. Cancer Ther. 16, 2598–2608. 10.1158/1535-7163.MCT-17-0386 28835386 PMC5670009

[B55] Gray E. S. Rizos H. Reid A. L. Boyd S. C. Pereira M. R. Lo J.. (2015). Circulating tumor DNA to monitor treatment response and detect acquired resistance in patients with metastatic melanoma. Oncotarget 6, 42008–42018. 10.18632/oncotarget.5788 26524482 PMC4747205

[B56] Gray E. S. Witkowski T. Pereira M. Calapre L. Herron K. Irwin D.. (2019). Genomic analysis of circulating tumor DNA using a melanoma-specific UltraSEEK oncogene panel. J. Mol. Diagn. 21, 418–426. 10.1016/j.jmoldx.2018.12.001 30731208

[B57] Greaves W. O. Verma S. Patel K. P. Davies M. A. Barkoh B. A. Galbincea J. M.. (2013). Frequency and spectrum of BRAF mutations in a retrospective, single-institution study of 1112 cases of melanoma. J. Mol. Diagn. 15, 220–226. 10.1016/j.jmoldx.2012.10.002 23273605 PMC5707183

[B58] Harlé A. Salleron J. Franczak C. Dubois C. Filhine-Tressarieu P. Leroux A.. (2016). Detection of BRAF mutations using a fully automated platform and comparison with high resolution melting, real-time allele specific amplification, immunohistochemistry and next generation sequencing assays, for patients with metastatic melanoma. PLoS ONE 11:e0153576. 10.1371/journal.pone.0153576 27111917 PMC4844167

[B59] Hayward N. K. Wilmott J. S. Waddell N. Johansson P. A. Field M. A. Nones K.. (2017). Whole-genome landscapes of major melanoma subtypes. Nature 545, 175–180. 10.1038/nature22071 28467829

[B60] Hintzsche J. D. Gorden N. T. Amato C. M. Kim J. Wuensch K. E. Robinson S. E.. (2017). Whole-exome sequencing identifies recurrent SF3B1 R625 mutation and comutation of NF1 and KIT in mucosal melanoma. Melanoma Res. 27, 189–199. 10.1097/CMR.0000000000000345 28296713 PMC5470740

[B61] Hodis E. Watson I. R. Kryukov G. V. Arold S. T. Imielinski M. Theurillat J.-P.. (2012). A landscape of driver mutations in melanoma. Cell 150, 251–263. 10.1016/j.cell.2012.06.024 22817889 PMC3600117

[B62] Hofman V. Ilie M. Long-Mira E. Giacchero D. Butori C. Dadone B.. (2013). Usefulness of immunocytochemistry for the detection of the BRAF(V600E) mutation in circulating tumor cells from metastatic melanoma patients. J. Invest. Dermatol. 133, 1378–1381. 10.1038/jid.2012.485 23303445

[B63] Hugo W. Zaretsky J. M. Sun L. Song C. Moreno B. H. Hu-Lieskovan S.. (2016). Genomic and transcriptomic features of response to anti-PD-1 therapy in metastatic melanoma. Cell 165, 35–44. 10.1016/j.cell.2016.02.065 26997480 PMC4808437

[B64] Ihle M. A. Fassunke J. König K. Grünewald I. Schlaak M. Kreuzberg N.. (2014). Comparison of high resolution melting analysis, pyrosequencing, next generation sequencing and immunohistochemistry to conventional sanger sequencing for the detection of p.V600E and non-p.V600E BRAF mutations. BMC Cancer 14:13. 10.1186/1471-2407-14-13 24410877 PMC3893431

[B65] Jahr S. Hentze H. Englisch S. Hardt D. Fackelmayer F. O. Hesch R. D.. (2001). DNA fragments in the blood plasma of cancer patients: quantitations and evidence for their origin from apoptotic and necrotic cells. Cancer Res. 61, 1659–1665. 11245480

[B66] Janku F. Vaishampayan U. N. Khemka V. Bhatty M. Sherman E. J. Tao J. (2018). Phase 1/2 precision medicine study of the next-generation BRAF inhibitor PLX8394. J. Clin. Oncol. 36, 2583–2583. 10.1200/JCO.2018.36.15_suppl.2583

[B67] Jennings L. J. Arcila M. E. Corless C. Kamel-Reid S. Lubin I. M. Pfeifer J.. (2017). Guidelines for validation of next-generation sequencing-based oncology panels: a joint consensus recommendation of the association for molecular pathology and college of american pathologists. J. Mol. Diagn. 19, 341–365. 10.1016/j.jmoldx.2017.01.011 28341590 PMC6941185

[B68] Jeong D. Jeong Y. Lee S. Lee H. Lee W. Kim H.. (2012). Detection of BRAFV600E mutations in papillary thyroid carcinomas by peptide nucleic acid clamp real-Time PCR: a comparison with direct sequencing. Korean J. Pathol. 46, 61–67. 10.4132/KoreanJPathol.2012.46.1.61 23109980 PMC3479705

[B69] Johansson P. Aoude L. G. Wadt K. Glasson W. J. Warrier S. K. Hewitt A. W.. (2015). Deep sequencing of uveal melanoma identifies a recurrent mutation in PLCB4. Oncotarget 7, 4624–4631. 10.18632/oncotarget.6614 26683228 PMC4826231

[B70] Johnson D. B. Frampton G. M. Rioth M. J. Yusko E. Xu Y. Guo X.. (2016). Targeted next generation sequencing identifies markers of response to PD-1 blockade. Cancer Immunol Res. 4, 959–967. 10.1158/2326-6066.CIR-16-0143 27671167 PMC5134329

[B71] Johnson D. B. Sosman J. A. (2015). Therapeutic advances and treatment options in metastatic melanoma. JAMA Oncol. 1, 380–386. 10.1001/jamaoncol.2015.0565 26181188

[B72] Jovanovic B. Egyhazi S. Eskandarpour M. Ghiorzo P. Palmer J. M. Scarrà G. B.. (2010). Coexisting NRAS and BRAF mutations in primary familial melanomas with specific CDKN2A germline alterations. J. Invest. Dermatol. 130, 618–620. 10.1038/jid.2009.287 19759551 PMC3665509

[B73] Jurkowska M. Gos A. Ptaszynski K. Michej W. Tysarowski A. Zub R. (2015). Comparison between two widely used laboratory methods in BRAF V600 mutation detection in a large cohort of clinical samples of cutaneous melanoma metastases to the lymph nodes. Int. J. Clin. Exp. Pathol. 8, 8487–8493. 26339422 PMC4555750

[B74] Kiniwa Y. Nakamura K. Mikoshiba A. Akiyama Y. Morimoto A. Okuyama R. (2018). Diversity of circulating tumor cells in peripheral blood: detection of heterogeneous BRAF mutations in a patient with advanced melanoma by single-cell analysis. J. Dermatol. Sci. 90, 211–213. 10.1016/j.jdermsci.2018.01.011 29426605

[B75] Knol A.-C. Vallée A. Herbreteau G. Nguyen J. Varey E. Gaultier A.. (2016). Clinical significance of BRAF mutation status in circulating tumor DNA of metastatic melanoma patients at baseline. Exp. Dermatol. 25, 783–788. 10.1111/exd.13065 27194447

[B76] Korn E. L. Liu P.-Y. Lee S. J. Chapman J.-A. W. Niedzwiecki D. Suman V. J.. (2008). Meta-analysis of phase II cooperative group trials in metastatic stage IV melanoma to determine progression-free and overall survival benchmarks for future phase II trials. J. Clini Oncol. 26, 527–534. 10.1200/JCO.2007.12.7837 18235113

[B77] Krauthammer M. Kong Y. Ha B. H. Evans P. Bacchiocchi A. McCusker J. P.. (2012). Exome sequencing identifies recurrent somatic RAC1 mutations in melanoma. Nat. Genet. 44, 1006–1014. 10.1038/ng.2359 22842228 PMC3432702

[B78] Kwong L. Chin L. (2010). The brothers RAF. Cell 140, 180–182. 10.1016/j.cell.2010.01.013 20141832

[B79] Lamy P.-J. Castan F. Lozano N. Montélion C. Audran P. Bibeau F.. (2015). Next-generation genotyping by digital PCR to detect and quantify the BRAF V600E mutation in melanoma biopsies. J. Mol. Diagn. 17, 366–373. 10.1016/j.jmoldx.2015.02.004 25952101

[B80] Lebofsky R. Decraene C. Bernard V. Kamal M. Blin A. Leroy Q. (2015). Circulating tumor DNA as a non-invasive substitute to metastasis biopsy for tumor genotyping and personalized medicine in a prospective trial across all tumor types. Mol. Oncol. 9, 783–790. 10.1016/j.molonc.2014.12.003 25579085 PMC5528781

[B81] Leon S. A. Shapiro B. Sklaroff D. M. Yaros M. J. (1977). Free DNA in the serum of cancer patients and the effect of therapy. Cancer Res. 37, 646–650. 837366

[B82] Lipson E. J. Velculescu V. E. Pritchard T. S. Sausen M. Pardoll D. M. Topalian S. L.. (2014). Circulating tumor DNA analysis as a real-time method for monitoring tumor burden in melanoma patients undergoing treatment with immune checkpoint blockade. J Immunother. Cancer 2:42. 10.1186/s40425-014-0042-0 25516806 PMC4267741

[B83] Lito P. Rosen N. Solit D. B. (2013). Tumor adaptation and resistance to RAF inhibitors. Nat. Med. 19, 1401–1409. 10.1038/nm.3392 24202393

[B84] Long G. V. Hauschild A. Santinami M. Atkinson V. Mandalà M. Chiarion-Sileni V.. (2017). Adjuvant dabrafenib plus trametinib in stage III BRAF-mutated melanoma. N. Engl. J. Med. 377, 1813–1823. 10.1056/NEJMoa1708539 28891408

[B85] Long G. V. Menzies A. M. Nagrial A. M. Haydu L. E. Hamilton A. L. Mann G. J.. (2011). Prognostic and clinicopathologic associations of oncogenic BRAF in metastatic melanoma. J. Clin. Oncol. 29, 1239–1246. 10.1200/JCO.2010.32.4327 21343559

[B86] Long G. V. Wilmott J. S. Capper D. Preusser M. Zhang Y. E. Thompson J. F.. (2013). Immunohistochemistry is highly sensitive and specific for the detection of V600E BRAF mutation in melanoma. Am. J. Surg. Pathol. 37, 61–65. 10.1097/PAS.0b013e31826485c0 23026937

[B87] Long-Mira E. Ilie M. Chamorey E. Leduff-Blanc F. Montaudié H. Tanga V.. (2018). Monitoring BRAF and NRAS mutations with cell-free circulating tumor DNA from metastatic melanoma patients. Oncotarget 9, 36238–36249. 10.18632/oncotarget.26343 30546839 PMC6281416

[B88] Lopez-Rios F. Angulo B. Gomez B. Mair D. Martinez R. Conde E.. (2013). Comparison of testing methods for the detection of BRAF V600E mutations in malignant melanoma: pre-approval validation study of the companion diagnostic test for vemurafenib. PLoS ONE 8:e53733. 10.1371/journal.pone.0053733 23326492 PMC3542342

[B89] Lovly C. M. Dahlman K. B. Fohn L. E. Su Z. Dias-Santagata D. Hicks D. J.. (2012). Routine multiplex mutational profiling of melanomas enables enrollment in genotype-driven therapeutic trials. PLoS ONE 7:e35309. 10.1371/journal.pone.0035309 22536370 PMC3335021

[B90] Lyu J. Song Z. Chen J. Shepard M. Song H. Ren G.. (2017). Whole-exome sequencing of oral mucosal melanoma reveals mutational profile and therapeutic targets: WES of OMM reveals genomic alterations. J. Pathol. 244:358–366. 10.1002/path.5017 29230811

[B91] Malicherova B. Burjanivova T. Grendar M. Minarikova E. Bobrovska M. Vanova B.. (2018). Droplet digital PCR for detection of BRAF V600E mutation in formalin-fixed, paraffin-embedded melanoma tissues: a comparison with Cobas® 4800, sanger sequencing, and allele-specific PCR. Am. J. Transl. Res. 10, 3773–3781. 10.1016/j.jmoldx.2017.11.009 30662627 PMC6291720

[B92] Mancini I. Simi L. Salvianti F. Castiglione F. Sonnati G. Pinzani P. (2019). Analytical evaluation of an NGS testing method for routine molecular diagnostics on melanoma formalin-fixed, paraffin-embedded tumor-derived DNA. Diagnostics 9:117. 10.3390/diagnostics9030117 31547467 PMC6787639

[B93] Marchant J. Mange A. Larrieux M. Costes V. Solassol J. (2014). Comparative evaluation of the new FDA approved THxID^TM^-BRAF test with high resolution melting and sanger sequencing. BMC Cancer 14:519. 10.1186/1471-2407-14-519 25037456 PMC4223712

[B94] Marconcini R. Galli L. Antonuzzo A. Bursi S. Roncella C. Fontanini G.. (2017). Metastatic BRAF K601E-mutated melanoma reaches complete response to MEK inhibitor trametinib administered for over 36 months. Exp. Hematol. Oncol. 6:6. 10.1186/s40164-017-0067-4 28344857 PMC5361706

[B95] Matallanas D. Birtwistle M. Romano D. Zebisch A. Rauch J. von Kriegsheim A.. (2011). Raf family kinases. Genes Cancer 2, 232–260. 10.1177/1947601911407323 21779496 PMC3128629

[B96] McEvoy A. Calapre L. Pereira M. Giardina T. Robinson C. Khattak M.. (2017). Sensitive droplet digital PCR method for detection of TERT promoter mutations in cell free DNA from patients with metastatic melanoma. Oncotarget 8, 78890–78900. 10.18632/oncotarget.20354 29108273 PMC5668006

[B97] McEvoy A. Wood B. Ardakani N. Pereira M. Pearce R. Cowell L.. (2018). Droplet digital PCR for mutation detection in formalin-fixed, paraffin-embedded melanoma tissues: a comparison with sanger sequencing and pyrosequencing. J. Mol. Diagn. 20, 240–252. 10.1016/j.jmoldx.2017.11.009 29305225

[B98] Menzies A. Haydu L. Visintin L. Carlino M. Howle J. Thompson J.. (2012). Distinguishing clinicopathologic features of patients with V600E and V600K BRAF-mutant metastatic melanoma. Clin. Cancer Res. 18, 3242–3249. 10.1158/1078-0432.CCR-12-0052 22535154

[B99] Michielin O. van Akkooi A. C. J. Ascierto P. A. Dummer R. Keilholz U. Committee E. G. (2019). Cutaneous melanoma: ESMO clinical practice guidelines for diagnosis, treatment and follow-up†. Ann. Oncol. 30, 1884–1901. 10.1093/annonc/mdz411 31566661

[B100] Moser J. C. Chen D. Hu-Lieskovan S. Grossmann K. F. Patel S. Colonna S. V. (2019). Real-world survival of patients with advanced BRAF V600 mutated melanoma treated with front-line BRAF/MEK inhibitors, anti-PD-1 antibodies, or nivolumab/ipilimumab. Cancer Med. 8, 7637–7643. 10.1002/cam4.2625 31677253 PMC6912019

[B101] Mouliere F. Chandrananda D. Piskorz A. M. Moore E. K. Morris J. Ahlborn L. B.. (2018). Enhanced detection of circulating tumor DNA by fragment size analysis. Sci. Transl. Med. 10:eaat4921. 10.1126/scitranslmed.aat4921food 30404863 PMC6483061

[B102] Ney J. Froehner S. Roesler A. Buettner R. Merkelbach-Bruse S. (2012). High-resolution melting analysis as a sensitive prescreening diagnostic tool to detect KRAS, BRAF, PIK3CA, and AKT1 mutations in formalin-fixed, paraffin-embedded tissues. Arch. Pathol. Lab. Med. 136, 983–992. 10.5858/arpa.2011-0176-OA 22938585

[B103] Palmieri G. Colombino M. Casula M. Manca A. Mandalà M. Cossu A.. (2018). Molecular pathways in melanomagenesis: what we learned from next-generation sequencing approaches. Curr. Oncol. Rep. 20:86. 10.1007/s11912-018-0733-7 30218391 PMC6153571

[B104] Panka D. J. Sullivan R. J. Mier J. W. (2010). An inexpensive, specific and highly sensitive protocol to detect the BrafV600E mutation in melanoma tumor biopsies and blood. Melanoma Res. 20, 401–407. 10.1097/CMR.0b013e32833d8d48 20679909 PMC2936688

[B105] Park C. Kim M. Kim M. J. Kim H. Ock C.-Y. Keam B.. (2019). Clinical application of next-generation sequencing-based panel to BRAF wild-type advanced melanoma identifies key oncogenic alterations and therapeutic strategies. Mol. Cancer Ther. 19, 1–8. 10.1158/1535-7163.MCT-19-0457 31826932

[B106] Pavlick A. C. Fecher L. Ascierto P. A. Sullivan R. J. (2019). Frontline therapy for BRAF-mutated metastatic melanoma: how do you choose, and is there one correct answer? Am. Soc. Clin. Oncol. Educ. Book 39, 564–571. 10.1200/EDBK_243071 31099689

[B107] Pearlstein M. V. Zedek D. C. Ollila D. W. Treece A. Gulley M. L. Groben P. A.. (2014). Validation of the VE1 immunostain for the BRAF V600E mutation in melanoma. J. Cutan. Pathol. 41, 724–732. 10.1111/cup.12364 24917033 PMC4167935

[B108] Pellegrini C. Cardelli L. Padova M. D. Nardo L. D. Ciciarelli V. Rocco T.. (2020). Intra-patient heterogeneity of BRAF and NRAS molecular alterations in primary melanoma and metastases. Acta Derm. Venereol. 100:adv00040. 10.2340/00015555-3382 31774543 PMC9128895

[B109] Pellegrini C. Di Nardo L. Cipolloni G. Martorelli C. De Padova M. Antonini A.. (2018). Heterogeneity of BRAF, NRAS, and TERT promoter mutational status in multiple melanomas and association with MC1R genotype: findings from molecular and immunohistochemical analysis. J. Mol. Diagn. 20, 110–122. 10.1016/j.jmoldx.2017.10.002 29061376

[B110] Pichler M. Balic M. Stadelmeyer E. Ausch C. Wild M. Guelly C.. (2009). Evaluation of high-resolution melting analysis as a diagnostic tool to detect the BRAF V600E mutation in colorectal tumors. J. Mol. Diagn. 11, 140–147. 10.2353/jmoldx.2009.080100 19213871 PMC2665863

[B111] Pinzani P. Salvianti F. Cascella R. Massi D. De Giorgi V. Pazzagli M.. (2010). Allele specific Taqman-based real-time PCR assay to quantify circulating BRAFV600E mutated DNA in plasma of melanoma patients. Clin. Chim. Acta 411, 1319–1324. 10.1016/j.cca.2010.05.024 20576522

[B112] Pollock P. M. Harper U. L. Hansen K. S. Yudt L. M. Stark M. Robbins C. M.. (2003). High frequency of BRAF mutations in nevi. Nat. Genet. 33, 19–20. 10.1038/ng1054 12447372

[B113] Qu K. Pan Q. Zhang X. Rodriguez L. Zhang K. Li H.. (2013). Detection of BRAF V600 mutations in metastatic melanoma: comparison of the cobas 4800 and sanger sequencing assays. J. Mol. Diagn. 15, 790–795. 10.1016/j.jmoldx.2013.07.003 23994118

[B114] Queirolo P. Spagnolo F. (2017). BRAF plus MEK-targeted drugs: a new standard of treatment for BRAF-mutant advanced melanoma. Cancer Metastasis Rev. 36, 35–42. 10.1007/s10555-017-9660-6 28299583

[B115] Rapisuwon S. Busam K. J. Parks K. Chapman P. B. Lee E. Atkins M. B. (2016). Discordance between cobas BRAF V600 testing and VE1 immunohistochemistry in a melanoma patient with bone marrow metastases. Am. J. Dermatopathol. 38, 687–689. 10.1097/DAD.0000000000000660 27541170 PMC5510737

[B116] Reid A. L. Freeman J. B. Millward M. Ziman M. Gray E. S. (2015). Detection of BRAF-V600E and V600K in melanoma circulating tumor cells by droplet digital PCR. Clin. Biochem. 48, 999–1002. 10.1016/j.clinbiochem.2014.12.007 25523300

[B117] Reiman A. Kikuchi H. Scocchia D. Smith P. Tsang Y. W. Snead D.. (2017). Validation of an NGS mutation detection panel for melanoma. BMC Cancer 17:150. 10.1186/s12885-017-3149-0 28228113 PMC5322598

[B118] Richter A. Grieu F. Carrello A. Amanuel B. Namdarian K. Rynska A.. (2013). A multisite blinded study for the detection of BRAF mutations in formalin-fixed, paraffin-embedded malignant melanoma. Sci. Rep. 3:1659. 10.1038/srep01659 23584600 PMC3625889

[B119] Robert C. Grob J. J. Stroyakovskiy D. Karaszewska B. Hauschild A. Levchenko E.. (2019). Five-year outcomes with dabrafenib plus trametinib in metastatic melanoma. N. Engl. J. Med. 381, 626–636. 10.1056/NEJMoa1904059 31166680

[B120] Rowe S. P. Luber B. Makell M. Brothers P. Santmyer J. Schollenberger M. D.. (2018). From validity to clinical utility: the influence of circulating tumor DNA on melanoma patient management in a real-world setting. Mol. Oncol. 12, 1661–1672. 10.1002/1878-0261.12373 30113761 PMC6165998

[B121] Rubinstein J. C. Sznol M. Pavlick A. C. Ariyan S. Cheng E. Bacchiocchi A.. (2010). Incidence of the V600K mutation among melanoma patients with BRAF mutations, and potential therapeutic response to the specific BRAF inhibitor PLX4032. J. Transl. Med. 8:67. 10.1186/1479-5876-8-67 20630094 PMC2917408

[B122] Sakaizawa K. Goto Y. Kiniwa Y. Uchiyama A. Harada K. Shimada S.. (2012). Mutation analysis of BRAF and KIT in circulating melanoma cells at the single cell level. Br. J. Cancer 106, 939–946. 10.1038/bjc.2012.12 22281663 PMC3305957

[B123] Sanmamed M. F. Fernández-Landázuri S. Rodríguez C. Zárate R. Lozano M. D. Zubiri L.. (2015). Quantitative cell-free circulating BRAFV600E mutation analysis by use of droplet digital PCR in the follow-up of patients with melanoma being treated with BRAF inhibitors. Clin. Chem. 61, 297–304. 10.1373/clinchem.2014.230235 25411185

[B124] Santiago-Walker A. Gagnon R. Mazumdar J. Casey M. Long G. V. Schadendorf D.. (2016). Correlation of BRAF mutation status in circulating-free DNA and tumor and association with clinical outcome across four BRAFi and MEKi clinical trials. Clin. Cancer Res. 22, 567–574. 10.1158/1078-0432.CCR-15-0321 26446943

[B125] Schreuer M. Meersseman G. Van Den Herrewegen S. Jansen Y. Chevolet I. Bott A.. (2016). Quantitative assessment of BRAF V600 mutant circulating cell-free tumor DNA as a tool for therapeutic monitoring in metastatic melanoma patients treated with BRAF/MEK inhibitors. J. Transl. Med. 14:95. 10.1186/s12967-016-0852-6 27095081 PMC4837559

[B126] Schvartsman G. Taranto P. Glitza I. C. Agarwala S. S. Atkins M. B. Buzaid A. C. (2019). Management of metastatic cutaneous melanoma: updates in clinical practice. Ther. Adv. Med. Oncol. 11:1758835919851663. 10.1177/1758835919851663 31205512 PMC6535734

[B127] Schwarzenbach H. Hoon D. S. B. Pantel K. (2011). Cell-free nucleic acids as biomarkers in cancer patients. Nat. Rev. Cancer 11, 426–437. 10.1038/nrc3066 21562580

[B128] Sensi M. Nicolini G. Petti C. Bersani I. Lozupone F. Molla A.. (2006). Mutually exclusive NRASQ61R and BRAFV600E mutations at the single-cell level in the same human melanoma. Oncogene 25, 3357–3364. 10.1038/sj.onc.1209379 16462768

[B129] Shinozaki M. O'Day S. J. Kitago M. Amersi F. Kuo C. Kim J.. (2007). Utility of circulating B-RAF DNA mutation in serum for monitoring melanoma patients receiving biochemotherapy. Clin. Cancer Res. 13, 2068–2074. 10.1158/1078-0432.CCR-06-2120 17404088 PMC2720029

[B130] Siegel R. L. Miller K. D. Jemal A. (2017). Cancer statistics, 2017. CA Cancer J. Clin. 67, 7–30. 10.3322/caac.21387 28055103

[B131] Snyder A. Makarov V. Merghoub T. Yuan J. Zaretsky J. M. Desrichard A.. (2014). Genetic basis for clinical response to CTLA-4 blockade in melanoma. N. Engl. J. Med. 371, 2189–2199. 10.1056/NEJMoa1406498 25409260 PMC4315319

[B132] Snyder M. W. Kircher M. Hill A. J. Daza R. M. Shendure J. (2016). Cell-free DNA comprises an *in vivo* nucleosome footprint that informs its tissues-of-origin. Cell 164, 57–68. 10.1016/j.cell.2015.11.050 26771485 PMC4715266

[B133] Spagnolo F. Boutros A. Tanda E. Queirolo P. (2019). The adjuvant treatment revolution for high-risk melanoma patients. Semin. Cancer Biol. 59, 283–289. 10.1016/j.semcancer.2019.08.024 31445219

[B134] Spagnolo F. Ghiorzo P. Orgiano L. Pastorino L. Picasso V. Tornari E.. (2015). BRAF-mutant melanoma: treatment approaches, resistance mechanisms, and diagnostic strategies. Onco. Targets. Ther. 8, 157–168. 10.2147/OTT.S39096 25653539 PMC4303458

[B135] Spittle C. Ward M. R. Nathanson K. L. Gimotty P. A. Rappaport E. Brose M. S.. (2007). Application of a BRAF pyrosequencing assay for mutation detection and copy number analysis in malignant melanoma. J. Mol. Diagn. 9, 464–471. 10.2353/jmoldx.2007.060191 17690212 PMC1975103

[B136] Stagni C. Zamuner C. Elefanti L. Zanin T. Bianco P. D. Sommariva A.. (2018). BRAF gene copy number and mutant allele frequency correlate with time to progression in metastatic melanoma patients treated with MAPK inhibitors. Mol. Cancer Ther. 17, 1332–1340. 10.1158/1535-7163.MCT-17-1124 29626128

[B137] Stroun M. Lyautey J. Lederrey C. Olson-Sand A. Anker P. (2001). About the possible origin and mechanism of circulating DNA apoptosis and active DNA release. Clin. Chim. Acta 313, 139–142. 10.1016/S0009-8981(01)00665-9 11694251

[B138] Swarup V. Moganty R. (2007). Circulating (cell-free) nucleic acids – a promising, non-invasive tool for early detection of several human diseases. FEBS Lett. 581, 795–799. 10.1016/j.febslet.2007.01.051 17289032

[B139] Tan Y. H. Liu Y. Eu K. W. Ang P. W. Li W. Q. Salto-Tellez M.. (2008). Detection of BRAF V600E mutation by pyrosequencing. Pathology 40, 295–298. 10.1080/00313020801911512 18428050

[B140] Tang H. Kong Y. Si L. Cui C. Sheng X. Chi Z.. (2018). Clinical significance of BRAFV600E mutation in circulating tumor DNA in Chinese patients with melanoma. Oncol. Lett. 15, 1839–1844. 10.3892/ol.2017.7529 29434880 PMC5777120

[B141] Tate J. G. Bamford S. Jubb H. C. Sondka Z. Beare D. M. Bindal N.. (2019). COSMIC: the catalogue of somatic mutations in cancer. Nucleic Acids Res. 47, D941–D947. 10.1093/nar/gky1015 30371878 PMC6323903

[B142] Tetzlaff M. T. Pattanaprichakul P. Wargo J. Fox P. P. Patel K. P. Estrella J. S.. (2015). Utility of BRAF V600E immunohistochemistry expression pattern as a surrogate of BRAF mutation status in 154 patients with advanced melanoma. Hum. Pathol. 46, 1101–1110. 10.1016/j.humpath.2015.04.012 26058727 PMC4515190

[B143] Thierry A. El Messaoudi S. Gahan P. Anker P. Stroun M. (2016). Origins, structures, and functions of circulating DNA in oncology. Cancer Metastasis Rev. 35, 347–376. 10.1007/s10555-016-9629-x 27392603 PMC5035665

[B144] Tschandl P. Berghoff A. S. Preusser M. Burgstaller-Muehlbacher S. Pehamberger H. Okamoto I.. (2013). NRAS and BRAF mutations in melanoma-associated nevi and uninvolved nevi. PLoS ONE 8:e69639. 10.1371/journal.pone.0069639 23861977 PMC3704624

[B145] Tsiatis A. C. Norris-Kirby A. Rich R. G. Hafez M. J. Gocke C. D. Eshleman J. R.. (2010). Comparison of sanger sequencing, pyrosequencing, and melting curve analysis for the detection of KRAS mutations: diagnostic and clinical implications. J. Mol. Diagn. 12, 425–432. 10.2353/jmoldx.2010.090188 20431034 PMC2893626

[B146] Uguen A. Talagas M. Costa S. Samaison L. Paule L. Alavi Z.. (2015). NRAS (Q61R), BRAF (V600E) immunohistochemistry: a concomitant tool for mutation screening in melanomas. Diagn. Pathol. 10:121. 10.1186/s13000-015-0359-0 26204954 PMC4513673

[B147] Valachis A. Ullenhag G. J. (2017). Discrepancy in BRAF status among patients with metastatic malignant melanoma: a meta-analysis. Eur J Cancer 81, 106–115. 10.1016/j.ejca.2017.05.015 28623774

[B148] Vallée A. Denis-Musquer M. Herbreteau G. Théoleyre S. Bossard C. Denis M. G. (2019). Prospective evaluation of two screening methods for molecular testing of metastatic melanoma: diagnostic performance of BRAF V600E immunohistochemistry and of a NRAS-BRAF fully automated real-time PCR-based assay. PLoS ONE 14:e0221123. 10.1371/journal.pone.0221123 31415669 PMC6695223

[B149] Valpione S. Gremel G. Mundra P. Middlehurst P. Galvani E. Girotti M. R.. (2018). Plasma total cell-free DNA (cfDNA) is a surrogate biomarker for tumor burden and a prognostic biomarker for survival in metastatic melanoma patients. Eur J Cancer. 88 1–9. 10.1016/j.ejca.2017.10.029 29175734 PMC5769519

[B150] Van Allen E. M. Miao D. Schilling B. Shukla S. A. Blank C. Zimmer L.. (2015). Genomic correlates of response to CTLA-4 blockade in metastatic melanoma. Science 350, 207–211. 10.1126/science.aad0095 26359337 PMC5054517

[B151] Vanni I. Alama A. Grossi F. Dal Bello M. G. Coco S. (2017). Exosomes: a new horizon in lung cancer. Drug Discov. Today 22, 927–936. 10.1016/j.drudis.2017.03.004 28288782

[B152] Volik S. Alcaide M. Morin R. D. Collins C. (2016). Cell-free DNA (cfDNA): clinical significance and utility in cancer shaped by emerging technologies. Mol. Cancer Res. 14, 898–908. 10.1158/1541-7786.MCR-16-0044 27422709

[B153] Wan P. T. C. Garnett M. J. Roe S. M. Lee S. Niculescu-Duvaz D. Good V. M.. (2004). Mechanism of activation of the RAF-ERK signaling pathway by oncogenic mutations of B-RAF. Cell 116, 855–867. 10.1016/S0092-8674(04)00215-6 15035987

[B154] Wilmott J. S. Johansson P. A. Newell F. Waddell N. Ferguson P. Quek C.. (2019). Whole genome sequencing of melanomas in adolescent and young adults reveals distinct mutation landscapes and the potential role of germline variants in disease susceptibility. Int. J. Cancer 144, 1049–1060. 10.1002/ijc.31791 30178487

[B155] Yancovitz M. Litterman A. Yoon J. Ng E. Shapiro R. L. Berman R. S.. (2012). Intra- and inter-tumor heterogeneity of BRAFV600EMutations in primary and metastatic melanoma. PLoS ONE 7:e29336. 10.1371/journal.pone.0029336 22235286 PMC3250426

[B156] Yao Z. Yaeger R. Rodrik-Outmezguine V. S. Tao A. Torres N. M. Chang M. T.. (2017). Tumors with class 3 BRAF mutants are sensitive to the inhibition of activated RAS. Nature 548, 234–238. 10.1038/nature23291 28783719 PMC5648058

[B157] Zhang C. Guan Y. Sun Y. Ai D. Guo Q. (2016a). Tumor heterogeneity and circulating tumor cells. Cancer Lett. 374, 216–223. 10.1016/j.canlet.2016.02.024 26902424

[B158] Zhang R. Nakahira K. Guo X. Choi A. M. K. Gu Z. (2016b). Very short mitochondrial DNA fragments and heteroplasmy in human plasma. Sci. Rep. 6:36097. 10.1038/srep36097 27811968 PMC5095883

[B159] Zhang T. Dutton-Regester K. Brown K. Hayward N. (2016c). The genomic landscape of cutaneous melanoma. Pigment Cell Melanoma Res. 29, 266–283. 10.1111/pcmr.12459 26833684

[B160] Zhou R. Shi C. Tao W. Li J. Wu J. Han Y.. (2019). Analysis of mucosal melanoma whole-genome landscapes reveals clinically relevant genomic aberrations. Clin. Cancer Res. 25, 3548–3560. 10.1158/1078-0432.CCR-18-3442 30782616

